# Synthesis of chitosan-encapsulated mono- and di-metallic (Zn/Ca) MOFs to modulate seed quality and antioxidant defense in *Chenopodium quinoa* under salinity stress

**DOI:** 10.1186/s12870-026-09734-w

**Published:** 2026-08-21

**Authors:** Noura E. Mahmoud, Reda M. Abdelhameed

**Affiliations:** 1 Biochemistry Unit, Genetic Resources Department, Desert Research Center, Cairo, Egypt; 2 Applied Organic Chemistry Department, Chemical Industries Research Institute, National Research Centre, Scopus affiliation ID 60014618, 33 EL Buhouth St., Dokki, Giza, 12622 Egypt

**Keywords:** Chitosan-MOFs composites, *Chenopodium quinoa* Willd., Salinity, Di-metallic MOFs, Biostimulants, Seed quality, Oxidative stress

## Abstract

Soil salinity is a major environmental constraint that severely limits the growth and nutritional yield of *Chenopodium quinoa* Willd. Although metal-organic frameworks (MOFs) show strong potential in agricultural settings, hybridizing them with biopolymers facilitates application under environmental conditions. This study aimed to develop a novel biostimulant by synthesizing chitosan-encapsulated mono-metallic (Zn-MOF) and di-metallic (Zn/Ca-MOF) frameworks and evaluating their efficacy in quinoa under saline field conditions. The composites were synthesized and characterized for their structural and chemical properties. Quinoa plants were cultivated under saline field conditions and treated with chitosan-MOF composites to assess physiological and biochemical responses, with a focus on nutritional value and the antioxidant defense system. Our findings revealed that application of the chitosan–MOF composites—most notably the bimetallic Zn/Ca formulation—substantially enhanced plant growth, biomass accumulation, and physiological performance under saline field conditions. Treatment with the composite enhanced the accumulation of key nutritional constituents in the seeds, specifically increasing the relative proportions of phytosterols, unsaturated fatty acids, and essential amino acids. Furthermore, application of the composite enhanced antioxidant enzyme activities (including SOD and POD), which was accompanied by a marked reduction in lipid peroxidation (45%) and hydrogen peroxide levels (57%). These physiological improvements corresponded to significant gains in plant biomass and seed quality parameters under saline field conditions. Overall, this study demonstrates that chitosan-encapsulated di-metallic frameworks serve as an effective, multi-functional strategy for enhancing salt tolerance in quinoa, offering a sustainable approach to improving crop resilience and food security in saline environments.

**Supplementary Information:**

The online version contains supplementary material available at 10.1186/s12870-026-09734-w.

## Introduction

The Chenopodiaceae family includes the quinoa plant (*Chenopodium quinoa* Willd.). Because of its multiple applications and health benefits, the Food and Agriculture Organization of the United Nations (FAO) has named it one of humanity’s most promising crops. It can also be used as a substitute to address issues with human nutrition [1]. As a pseudocereal of high nutritional value, quinoa provides a rich matrix of proteins, lipids, dietary fiber, vitamins, and minerals, characterized by a complete and balanced essential amino acid profile. In addition to its primary macronutrient and micronutrient density, quinoa synthesizes diverse bioactive phytochemicals, notably phytosterols, phytoecdysteroids, and saponins. Clinical and preclinical studies suggest that these functional components contribute to the positive modulation of metabolic, cardiovascular, and gastrointestinal health [2]. Furthermore, quinoa may lower the risk of cancer, diabetes, obesity, and cardiovascular disease [3–6]. The FAO proclaimed 2013 the “International Year of Quinoa” due to its high nutritional value, and it has been said that this is one of the cultures created to provide food security in the twenty-first century [7, 8]. Using the Food Ex 2 food classification and description system, the European Food Safety Authority (EFSA) has also included quinoa on the list of functional foods/products with health claims (A000R) [9]. The combined hues of the pericarp and episperm determine the color of quinoa seeds, which can range from white to gray to black, yellow, and red [10]. Quinoa pigments called betalains have anti-inflammatory and antioxidant qualities that are comparable to those of phenolic substances [11, 12]. In addition to calcium oxalate, the outer coats of quinoa seeds contain substances such as tannins, phytates, saponins, and protease inhibitors [13–15]. Specifically, quinoa seeds contain chemicals called saponins, which might vary in concentration depending on the variety being researched [16]. Quinoa’s enormous genetic variety allows it to thrive in even the most unfavorable geographic and climatic situations. Given that deserts make up almost one-third of the Earth’s geographical area, sustainable desert farming has become a crucial area of study in agricultural research within the past ten years.

Arid and semi-arid regions face severe agricultural constraints due to concurrent abiotic stresses, including soil/water salinity, high temperatures, and low soil fertility. To stabilize and advance sustainable agriculture in these areas, identifying stress-tolerant crops is vital. Quinoa (*Chenopodium quinoa* Willd.) has become an ideal candidate and is widely cultivated under brackish irrigation in the Middle East and North Africa (MENA) region due to its halophytic (salt-tolerant) characteristics [17]. An evaluation by the International Center for Biosaline Agriculture (ICBA) tested 11 quinoa varieties in sandy soils under low salinity (640 ppm) and high salinity (9600 ppm) regimes. High salinity resulted in a 30% reduction in seed yield, a 20% decline in total biomass, and a 6% decrease in thousand-grain weight (TGW) [18]. A high sodium concentration has a negative impact on the acquisition of vital nutrients in the second phase because it competitively limits the uptake of potassium and calcium [19] and affects numerous vital metabolic pathways that are essential to plant growth [20] and ultimately the quality of the plants [21].

Chitosan is a natural, modified, and positively charged (cationic) polysaccharide derived from chitin, which is found in the shells of shellfish (shrimp, crabs, and lobsters) and some fungal cell walls [22]. Chitosan treatment stimulates chlorophyll synthesis and photosynthetic activity [23], which supports enhanced symbiotic nitrogen fixation in leguminous species [24]. By mitigating environmental stress and optimizing nutrient and water absorption, chitosan application consistently improves plant growth and yield parameters [25]. Furthermore, it was discovered that applying chitosan spray to salt-stressed plants has effects on plant growth regulators such as abscisic acid and jasmonic acid [26]. Additionally, chitosan and salinity had an additive influence on the level of total soluble solids, proline, and carotenoids [27].

Metal-organic frameworks (MOFs) are a class of porous crystalline materials synthesized via the self-assembly of metal ions or clusters with organic linkers. These hybrid networks possess highly customizable pore dimensions, exceptionally high specific surface areas, abundant active sites, and tailorable surface chemistry. Furthermore, their high chemical stability and functional versatility afford substantial adsorption capacities and modular functionality across diverse applications [28]. Furthermore, the broad diversity of metal centers, coordination bonds, and functional groups within MOF architectures enables tailored host–guest interactions. Deciphering these intermolecular forces allows for the rational design of frameworks optimized for targeted molecular binding and enhanced adsorption capacity [29]. MOFs have attracted significant interest in agricultural applications due to their hybrid inorganic–organic architectures, which combine the structural versatility of organic linkers with the catalytic and chemical functionality of metal nodes. Integrating organic and inorganic chemistry within a single crystalline lattice allows for precise control over framework composition, degradation pathways, and guest-molecule interactions, presenting new opportunities for targeted crop protection and smart nutrient delivery [30].

Similarly, the application of zeolitic imidazolate framework-8 (ZIF-8) and magnesium oxide-loaded ZIF-8 (MgO@ZIF-8), combined with *Aloe vera* extracts, was evaluated on quinoa growth, yield, biochemical profiles, and molecular responses under salinity [31]. Among the tested cultivars, the Chipaya and Line 14 genotypes exhibited superior biomass accumulation, photosynthetic pigment retention, enhanced antioxidant enzyme activity, and higher seed yield.

Calcium (Ca^2+^) and zinc (Zn^2+^) serve distinct yet essential functions in plant growth and development. Calcium predominantly acts as a structural component, cross-linking pectins to stabilize cell walls and plasma membranes, while also functioning as a pivotal second messenger in signal transduction cascades during environmental stress responses. In contrast, zinc is an essential trace element required for protein synthesis, enzymatic activation, chlorophyll biosynthesis, and gene regulation through zinc-finger motifs.

Consequently, zinc deficiency impairs metabolic efficiency, resulting in yield penalties and reduced crop nutritional quality. To enhance zinc use efficiency (ZUE), recent research has focused on elucidating the transport mechanics of uptake and translocation, organic acid-mediated chelation, and the architectural optimization of root systems [32]. The authors concluded that optimizing root architecture, applying biostimulants, and deploying engineered nanomaterials to enhance zinc biofortification represent viable strategies to improve zinc use efficiency (ZUE), preserve crop nutritional quality, and advance sustainable agricultural systems.

Conversely, calcium (Ca^2+^) is an essential macronutrient required for plant growth and development across both basal and stress conditions. It executes a dual physiological role: functioning as a structural element that cross-links cell walls and stabilizes plasma membranes, and operating as a ubiquitous second messenger in signal transduction pathways governing environmental adaptation. Generating these stimulus-specific intracellular signals depends strictly on the maintenance of tight cytosolic Ca^2+^ homeostasis. While the detailed mechanisms regulating calcium uptake, transport, cellular homeostasis, and signal transduction have been extensively reviewed [33], the positive growth responses observed in the present study following calcium and zinc co-application may be attributed to these structural and signaling functions, although the direct physiological pathways warrant further empirical verification [33].

Recent research underscores the importance of the CCO gene family in hormone-mediated stress signaling and the role of biosensing technologies in rapid pathogen detection. Concurrently, the development of biogenic ZnO-NPs and Zn-Lys chelates has provided eco-friendly avenues for enhancing antioxidant defenses and alleviating heavy metal toxicity. Furthermore, leveraging natural symbioses such as arbuscular mycorrhizal fungi ensures optimized mineral nutrition and soil restoration, collectively strengthening crop resilience in challenging environments [34–37].

Current advancements in sustainable agriculture emphasize multi-targeted strategies to combat abiotic and biotic pressures. Recent studies demonstrate that green-synthesized nanoparticles [38–40] and phytohormonal interventions [41] significantly improve crop resilience by maintaining ion homeostasis and enhancing photosynthetic efficiency [42]. The emerging synergy between nano-carriers and hormonal signaling [43, 44], coupled with soil-level amendments to restore microbial diversity [45], represents a holistic approach to ensuring food security in increasingly marginal and saline environments.

Encapsulating the di-metallic framework within chitosan yields a hybrid composite with new functional groups that affect plant growth under salt stress. Furthermore, this composite provides sustained nutrient delivery that mitigates oxidative damage under saline conditions more effectively than traditional bulk salts. This work advances the application of reticular materials in agriculture, offering a waste-valorized approach to enhance crop tolerance under environmental stress.

The general aim of this study is to evaluate the synergistic potential of chitosan-modified di-metallic metal-organic frameworks (Chs@Zn/Ca-BDC) as a “smart” fertilizer and biostimulant for enhancing the resilience and productivity of quinoa (*Chenopodium quinoa* Willd.) under saline field conditions. The first aim was to develop the mono-metallic (Zn-BDC, Ca-BDC) and di-metallic (Zn/Ca-MOFs) modified with a chitosan matrix. The second aim was to evaluate physiological and yield responses. The third aim was to assess antioxidant and metabolic defense by quantifying lipid peroxidation (MDA), hydrogen peroxide (H_2_O_2_), and the activity of key antioxidant enzymes (SOD and POD). The fourth aim was to evaluate qualitative changes using Scanning Electron Microscopy (SEM), focusing on leaf stomatal morphology and the visual reinforcement of stem vascular tissues (xylem and cambium) following treatment application.

## Materials and methods

### Chemicals

Zinc chloride (ZnCl_2_), 2-aminoterphthalic acid (H_2_BDC-NH_2_), and calcium chloride (CaCl_2_) were purchased from Sigma Aldrich, Germany. Ethanol, methanol, *N*,* N*-dimethylformamide (DMF), and triethylamine were procured from Fluka Chemicals. All the chemicals were of analytical grade and used as received.

The materials used in the current study were named as Ca-Zn-BDC-N = Chs, meaning mixed metal from Ca and Zn was used to obtain the MOF system and modified with chitosan, NH_2_ = ligand without modification but, N = Chs = encapsulation.

### Synthesis of Ca-BDC-NH_2_

A mixture solution was prepared by dissolving 3.32 g of CaCl_2_ (29.91 mmol) and 5.41 g (29.89 mmol) of H_2_BDC-NH_2_ in 190 mL DMF. 0.5 mL of triethylamine was added to the mixed solution under ultrasonication. The precipitate appeared, showing the formation of MOFs. After 20 min of ultrasonication, the precipitate was collected by centrifugation, washed with ethanol, and dried at 50 °C for 10 days in a hot air oven to achieve the final MOFs.

### Synthesis of Zn-BDC-NH_2_

Zn-BDC-NH_2_ was synthesized via our modified method, H_2_BDC-NH_2_ (10.86 g, 60 mmol) was dissolved in 3 mL *N*,*N*-dimethylformamide (DMF). A solution of ZnCl_2_ (8.18 g,60 mmol) was dissolved in DMF (2 mL) and quickly added to the above mixture under stirring, and the whole was further stirred for 30 min. Trimethylamine (0.5 mL) was added to the above mixture, and the product was collected by centrifugation at 15,000 rpm for 20 min. The as-synthesized sample was washed with DMF (15 × 3 mL, each day) for 3 days and methanol (15 × 3 mL, each day) for 3 days.

### Synthesis of Ca-Zn-BDC-NH_2_

In a typical synthesis of Ca-Zn − BDC-NH_2_, 2.172 g of H_2_BDC-NH_2_ (12 mmol) was added to a mixture of 3 mL ethanol and 30 mL of DMF. In a separate experiment, 0.666 g of CaCl_2_ (6 mmol) and 6 mmol of ZnCl_2_ (0.818 g) were dissolved in 60 mL of DMF, then it was slowly added to the first mixture, followed by the addition of 0.5 mL of triethylamine (4 mmol). The mixture was then sonicated for 30 min to obtain a white precipitate. The solid was then washed with ethanol three times each, followed by drying in an oven at 100 °C.

### Preparation of MOF-Chs composite

The MOFs-Chs composite was prepared as follows:Ca-BDC-N = Chs was prepared by dissolving 4 g of Chs in 150 mL of 1% (v/v) acetic acid, then Ca-BDC-NH_2_ (0.5 g) was added to the above solution, followed by the addition of a cross-linking agent of 0.1 M glutaraldehyde. The mixture was then stirred for 24 h, and the precipitate was separated and oven-dried at 50 °C. Zn-BDC-N = Chs and Ca-Zn-BDC-N = Chs were prepared using the same previous method except for adding (0.5 g) Zn-BDC-NH_2_ and (0.5 g) Ca-Zn-BDC-NH_2_, separately, rather than Ca-BDC-NH_2_; the solids were dried and stored until use.

### Instruments and equipment

X-ray diffraction of powdered MOFs was obtained on an Ultima IV X-ray diffractometer (Rigajku, Japan) with Cu Kα and a Ni filter, where the scanning speed was set to 3° min^− 1^. A Hitachi S-4300 scanning electron microscope was used to obtain the scanning electron microscopy (SEM) images after coating with a gold-platinum alloy by ion-sputtering (E-1048 Hitachi ion sputter). An FTIR spectrometer (Cary670, Agilent) was used for recording the Fourier-transform infrared (FTIR) spectrum of MOFs after pelletization with KBr.

### Experimental design, sampling strategy, and sample preparation

Field experiments were conducted over two consecutive seasons, 2024–2025 and 2025–2026, at the Ras Sudr Research Station, South Sinai Governorate (a saline field area), Egypt. WAD8-1RE (1RE) and WAD8-3YE (3YE), two quinoa genotypes purchased from the Desert Research Center in Egypt, were the subjects of the study. In Ras Sudr, 1RE and 3YR quinoa seeds were planted in the late first third of November at a rate of roughly 3 Kg feddan^− 1^ (7 kg ha^− 1^). The field experiment was conducted using a split-plot design with three independent biological replicates (*n* = 3). Each experimental unit was defined as an individual field plot measuring 3 m x 3 m containing approximately 50 quinoa plants. Three replicates of each seed were planted in rows, 50 cm apart between rows. Before being planted, the seeds were steeped in water for three h. They were planted 20 cm apart in holes in rows, with roughly 6–8 seeds buried in each hole, 2.5 cm below the soil’s surface. After that, there were only two plants in each hole. Every three days, the quinoa was consistently drip-irrigated at a rate of 4 L per hour for 30 min and fertilized regularly. When preparing the soil for cultivation, 10 m^3^ feddan^− 1^ of organic fertilizer was applied. The following method was used to apply mineral fertilizer (N-P-K) at a rate of 20:20:50 kg feddan^− 1^: four equal dosages of nitrogen fertilizer should be applied. The first dose was administered after planting, the second 20 days after planting, the third 40 days later, and the fourth 60 days later. Two doses of phosphate fertilizer were added, first when the soil was being prepared for planting, and the second dose was applied 30 days following planting. Three dosages of potassium fertilizer were applied: one before planting, one twenty-five days later, and one seventy-five days thereafter. The chemical analysis of soil and water was done where the pH of the soil was 7.31, EC = 3.50 dS m^− 1^ and water pH = 6.9, EC = 15.09 dS m^− 1^ and mechanical dry sieving properties in the table (S1), the soil is sandy texture according to soil analysis in the same table.

### Foliar application protocol and material dosage

To ensure uniform coverage and avoid particle agglomeration on the leaf surfaces, the formulations (Ca-BDC-NH_2_, Zn-BDC-NH_2_, Chs, and their respective Schiff-base composites) were prepared as highly dispersed aqueous suspensions using a rigorous multi-step dispersion protocol before foliar application. For each treatment, 20 ppm of the synthesized material was suspended in deionized water. To break up loose agglomerates and ensure true dispersion, the crude suspensions were subjected to probe sonication using an ultrasonic processor equipped with a titanium microtip. Sonication was performed for 30 min at an operating frequency of 20 kHz with an amplitude of 60%. To prevent thermal degradation or premature structural breakdown of the MOF-Chitosan hybrid structures due to localized ultrasonic heating, the sample containers were kept immersed in a circulating ice-water bath throughout the entire sonication process. Sonication was delivered using a pulsed cycle (30 s on, 10 s off). The pH of the foliar spray suspensions is a critical factor influencing both material surface charge and plant physiological compatibility. Immediately post-sonication, the pH of all suspensions was checked. The values were carefully adjusted to a physiologically compatible range of 6.5 to 6.8 using highly dilute aqueous solutions of sodium hydroxide (0.05 M NaOH) or hydrochloric acid (0.05 M HCl).

The suspended solutions were sprayed on the two quinoa varieties after 25 and 50 days from planting in the morning with 400 liters of Ca-BDC-NH_2_, Zn-BDC-NH_2_, chitosan (Chs), Ca-BDC-N = Chs, Zn-BDC-N = Chs, Ca-Zn-BDC-NH_2_, and Ca-Zn-BDC-N = Chs. Foliar application was performed at an empirically selected concentration of 20 ppm, guided by working ranges reported for related biopolymeric and metal composites under saline field conditions [46–49].

### Gathering plant samples

Sixty days post-cultivation (coinciding with the full flowering stage), five plants were randomly harvested per replicate for each treatment. Plant height and fresh biomass were recorded immediately upon sampling. To determine dry biomass, plant tissues were oven-dried at 65 °C until reaching a constant weight. Fresh tissue samples were stored at -20 °C for subsequent biochemical analyses, which included the determination of photosynthetic pigment content, malondialdehyde (MDA), hydrogen peroxide (H_2_O_2_), total antioxidant capacity, and the activities of antioxidant enzymes, specifically peroxidase (POD) and superoxide dismutase (SOD). At 120 days post-cultivation, mature plants were harvested to quantify seed and straw yields. Inflorescences were air-dried at ambient room temperature before seed threshing. Chromatographic methods were subsequently employed to identify and profile active bioactive compounds, including fatty acids and amino acids.

### Biochemical analysis

#### Photosynthetic pigment determination

Fresh quinoa leaves were ground with 85% acetone to extract the carotenoids and chlorophyll *a* and *b*, which were then measured using spectrophotometry. The Wettstein formula was used to determine the amounts of carotenoids, chlorophyll a, and b. The final pigment content was reported in mg g^− 1^ of fresh leaf weight [50].

#### Analysis of malondialdehyde content

With some changes, the final product, malondialdehyde (MDA), was estimated to determine the levels of lipid peroxidation in the test samples [51]. One mL of 0.1% (w/v) trichloroacetic acid (TCA) was used to homogenize a 0.5 g sample of quinoa leaves. After centrifuging the homogenate for 15 min at 15,000 rpm, 0.5 g of thiobarbituric acid was dissolved in a 20% w/v trichloroacetic acid solution. Two mL of the combination were then added to 0.5 mL of the liquid that covered the leaf sample. After 30 min of heating at 95 °C in a water bath, the mixture was incubated for 5 min in an ice bath. The overlaying liquid was measured at 532 nm following centrifugation. Additionally, each sample’s nonspecific absorbance value was measured at 600 nm and deducted from the absorbance measured at 532 nm. Using the MDA standard curve, the concentration of the added MDA-TBA was computed and converted to µg g^− 1^ fresh weight.

#### Analysis of hydrogen peroxide content

The hydrogen peroxide content of fresh quinoa leaves cultivated in saline circumstances was measured using the oxidation of potassium iodide by H_2_O_2_ in acidic media as a basis for hydrogen peroxide levels [52]. The color concentration was ascertained by spectrophotometric analysis. The absorbance of the top liquid was measured at a wavelength of 390 nm. The concentration of hydrogen peroxide was determined using a standard curve.

#### Antioxidant capacity

The following is how DPPH (2,2-diphenyl-1-picrylhydrazyl) was measured:

0.5 mL of fresh leaf quinoa methanolic extract was combined with 2 mL of a 0.004% DPPH solution in 80% methanol. After 30 s of shaking, the reaction mixture was allowed to sit at room temperature in the dark for 30 min. Using spectrophotometry, the combined absorption wavelength was determined to be 517 nm [53]. The following formula was used to determine the capacity to scavenge the free radical:

$$\begin{aligned}\mathrm{RSC}\;(\%)\;&=\;100\;\mathrm{x}\;(\text{Abs blank}\;\\&-\;\text{Abs sample}\;/\text{Abs blank})\end{aligned}$$

Where (RSC %) = Radical scavenging capacity (%), Abs blank was the absorbance of methanolic DPPH without the plant sample.

#### Patterning of the superoxide dismutase isoenzyme utilizing the Native PAGE technique

Superoxide dismutase (SOD), an antioxidant enzyme that measures the quantity and activity of bands, can be measured using basic techniques defined by the Native PAGE protocol [54]. Superoxide radicals are changed into hydrogen peroxide and molecular oxygen by superoxide dismutase molecules. Using [54]. Nitroblue tetrazolium reduction is employed as an indication of O.^−^_2_ production, xanth-xanthine oxidase is used to generate O.^−^_2_, and SOD was isolated from quinoa leaves and separated using native polyacrylamide gel electrophoresis (PAGE). Following the appearance of bands, the gel was exposed to fluorescent light, which caused the gel to turn blue and should have produced clear bands. A specialized computer application was used to determine the sample’s band intensity (Gel Analyzer 2010).

### Analysis of peroxidase activity

Five mL of 0.05 M sodium phosphate buffer (pH 7.0) was used to homogenize roughly 0.5 g of fresh quinoa leaves. After centrifuging the homogenate at 20,000 rpm for 15 min at 4 °C, the supernatant-which represented the crude enzyme source-was stored at -20 °C until it was needed. According to Worthington Biochemical Corp., peroxidase was measured using the *O*-Dianisidine technique [55]. 0.1 mL of the enzyme was added and mixed with 1.5 mL of 0.01 M phosphate buffer (pH 6.0) and 0.01 mL of 1% *O*-Dianisidine (1% in absolute methanol). After mixing, 0.1 mL of 0.3% H_2_O_2_ was added, and the spectrophotometer recorded the increase in absorbance at 460 nm for three minutes as (Δ Abs 460 / mg fresh weight / 3 min).

#### Extraction and analysis of amino acids

Amino acid profiling was investigated in quinoa seed according to the previous method [56], with slight modifications. Briefly, 1.0 g of ground quinoa seed powder was defatted with diethyl ether. A 0.4 g portion of the defatted sample was subjected to acid hydrolysis using 5 mL of 6 N HCl at 110 °C for 24 h. The hydrolysate was filtered through Whatman filter paper and evaporated to dryness in a water bath at 70 °C. The resulting residue was reconstituted in sample dilution buffer and filtered through a 0.22 μm membrane filter. Prepared samples were stored in sealed vials at -20 °C until chromatographic analysis. Amino acid quantification was performed using an automated amino acid analyzer (SYKAM) equipped with an LCAKO6Na cation-exchange column (150 * 4.6 mm). Peaks were identified, and concentrations were calculated relative to external amino acid standards using Clarity software.

#### Lipid extraction and fatty acid analysis

Lipids were extracted from crushed quinoa seeds using a modified Soxhlet extraction protocol [57]. Briefly, 20 g of ground seeds were placed in a Soxhlet extractor and subjected to continuous extraction using diethyl ether for 6 h. The solvent was subsequently removed under reduced pressure using a rotary evaporator to yield the refined seed oil. Fatty acid methyl esters (FAMEs) were prepared according to [58]. A 2 mL aliquot of the extracted oil was subjected to alkali-catalyzed transesterification with methanol in the presence of 2 M potassium hydroxide. The resulting FAMEs were partitioned into hexane before chromatographic analysis.

An Agilent Technologies GC model 7890B fitted with a flame ionization detector at the Central Laboratories Network, National Research Center, Cairo, Egypt, was used to estimate the fatty acid composition using GC-FID in accordance with the Official Method [59]. A Zebron ZB-FAME column (60 m × 0.25 mm inner diameter × 0.25 μm film thickness) was used for the separations. The following temperature program was used for the analyses: 100 °C for 3 min, rising at 2.5 °Cminutes^-1^ to 240 °C and holding for 10 min. The hydrogen was used as the carrier gas, and the flow rate was 1.8 mL min^− 1^ in a 1:50 splitting mode, with an injection volume of 1 µL. At 250 °C and 285 °C, respectively, the injector and detector (FID) were maintained.

#### Unsaponifiable extraction and analysis

Weigh accurately 2.0 g of extracted quinoa seed oil into a round-bottom flask. Add 50 mL of 2 N ethanolic KOH solution. Attach a reflux condenser and heat in a water bath at 80 °C for 60 min with continuous stirring until saponification is complete (clear, homogeneous liquid). Allow the flask to cool to room temperature. Add 50 mL of distilled water to prevent emulsion formation and dissolve the potassium soaps. Transfer the mixture to a 250 mL separator funnel. Add 50 mL of diethyl ether and shake vigorously for 1–2 min, periodically releasing pressure. Allow the phases to separate. Collect the lower aqueous phase (soaps) in a second funnel and drain the upper organic layer (containing the USF) into a clean flask. Extract the aqueous layer twice more with 30 mL portions of organic solvent. Combine all organic phases in the separatory funnel. Wash the combined organic extract with 30 mL portions of distilled water until the washings test neutral to the phenolphthalein indicator (removing residual alkali and soaps). Pass the washed organic phase through a filter paper filled with anhydrous Na₂SO₄. Concentrate the dried solution using a rotary evaporator under reduced pressure at 40 °C until dry. Then store the dry residue at -20 °C before derivatization. Dissolve 20 mg of the dry USF residue in 100 µL of dry pyridine (acid scavenger/solvent) in a 2 mL glass autosampler vial with a PTFE-lined cap. Add 100 µL of BSTFA (N, O-bis (trimethylsilyl) trifluoroacetamide) with 1% TMCS (trimethylchlorosilane). Seal the vial and heat in a heating block at 60–70 °C for 30 min. Allow the vial to cool to room temperature. Dilute with high-purity n-hexane to a final concentration suitable for injection and transfer to a GC-Mass autosampler insert [60]. The GC-MS system (Agilent Technologies) was equipped with a gas chromatograph (7890B) and mass spectrometer detector (5977A) at Central Laboratories Network, National Research Centre, Cairo, Egypt. The GC was equipped with an HP-5MS column (15 m x 0.25 mm internal diameter and 0.25 μm film thickness). Analyses were carried out using hydrogen as the carrier gas at a flow rate of 1.1 mL min^− 1^ at a splitless injection volume of 1.0 µL and the following temperature program: 40 °C for 1 min; rising at 10 °C min^− 1^ to 200 °C and held for 1 min; rising at 20 °C min^− 1^ to 220 °C and held for 1 min; rising at 30 °C /min to 300 °C and held for 3 min. The injector and detector were held at 280 °C, 300 °C. Mass spectra were obtained by electron ionization (EI) at 70 eVusing a spectral range of m/z 50–800 and solvent delay of 3.10 min. The mass temperature was 230 °C and Quad 150 °C. Identification of different constituents was determined by comparing the fragmentation pattern with those stored in the Wiley and NIST Mass Spectral Library data.

### Sample prepared for microscopic analysis

A scanning electron microscope (SEM) specimen was prepared using glutaraldehyde as the fixative. The specimen was fixed by immersing it completely in a 5% glutaraldehyde solution overnight, followed by thorough rinsing with distilled water. It was then placed in a graded ethanol solution (75%, 95%, and 100%) for 15 min at each concentration and finally dried at room temperature. The dried specimen was securely mounted on the SEM base, coated with gold, and then imaged [61]. For Scanning Electron Microscopy (SEM) analysis, three independent plants were randomly selected per treatment as biological replicates (*n* = 3), and two fully expanded leaves or stem segments were collected per plant. From each tissue sample, multiple fields of view were screened. The images presented in this manuscript were selected as representative structures based on the consistent trends observed across all replicates. No direct quantitative morphometric measurements (such as stomatal aperture diameter or vessel thickness) were executed; thus, SEM data are presented as qualitative morphological descriptions.

### Statistical analysis

Statistical analyses were performed using MSTAT-C software (version 2.10) [62]. Data from two consecutive growing seasons (2024–2025 and 2025–2026) were analyzed using a mixed-model analysis of variance (ANOVA) structured as a split-plot design. Quinoa genotypes (3YE and 1RE) were assigned to the main plots, while the external application treatments (8 formulations) served as the subplots. Genotypes, external applications, and their interactions were designated as fixed effects, whereas experimental blocks (replications) and the block X genotype interaction were treated as random effects to construct the appropriate error terms for the split-plot framework.

Before pooling the data across seasons, homogeneity of variance was confirmed using Levene’s test (*p* > 0.05). A combined ANOVA incorporating season as a factor demonstrated that neither the main effect of season nor its interactions with genotype or treatment were statistically significant (*p* > 0.05), reflecting the uniform climatic conditions between the two years. Consequently, data from both seasons were averaged and pooled for the final integrated analysis. Pooling under homogeneous environmental conditions broadens the scope of statistical inference and enhances analytical power without compromising accuracy [63–65]. Salinity was excluded from the model due to its stability throughout the study period.

## Results

### Description of synthesized fertilizer

**Fig. 1:**
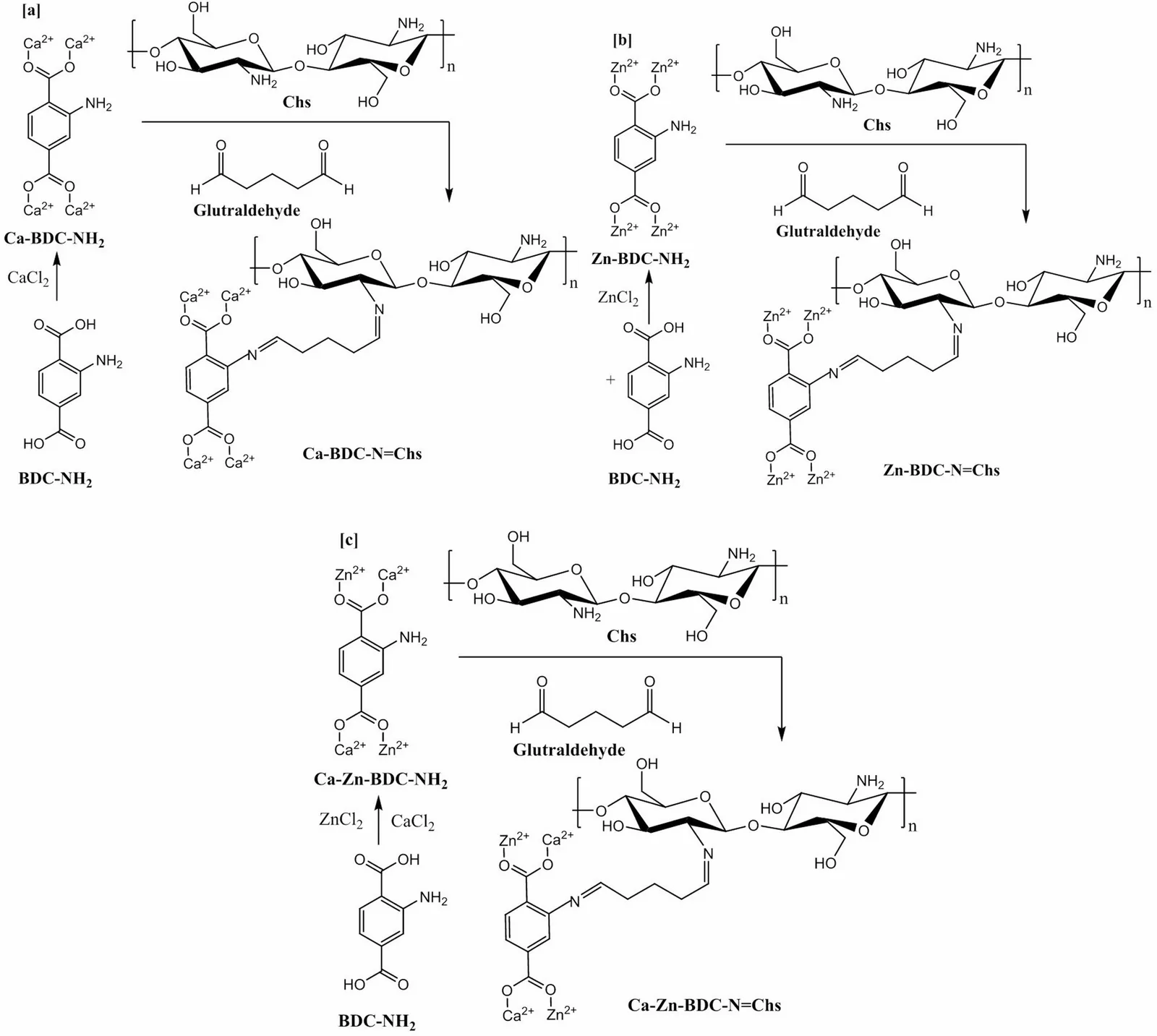
Synthetic protocol for preparation of Ca-BDC-NH_2_, Zn-BDC-NH_2_, Ca-Zn-BDC-NH_2_, and its chelating with chitosan

Figure 1 outlines the synthesis of mono-metallic Zn-BDC and di-metallic Zn/Ca-BDC, followed by their encapsulation in a chitosan (Chs) matrix. The process typically involves a solvothermal/hydrothermal synthesis of the MOF crystals, followed by PSM with biopolymer Chs. Mono-metallic Zn-BDC or Ca-BDC were synthesized based on zinc chloride or CaCl_2_ and 2-aminoterephthalic acid. It was synthesized using a mixed metal solution by dissolving Zn and Ca salts in a specific molar ratio (1:1). Chitosan was encapsulated with MOF via PSM surface modification. It was done by dispersing the MOF powder in a very dilute chitosan solution to allow the amino groups of the chitosan to coordinate with the metal sites on the MOF surface.

**Fig. 2:**
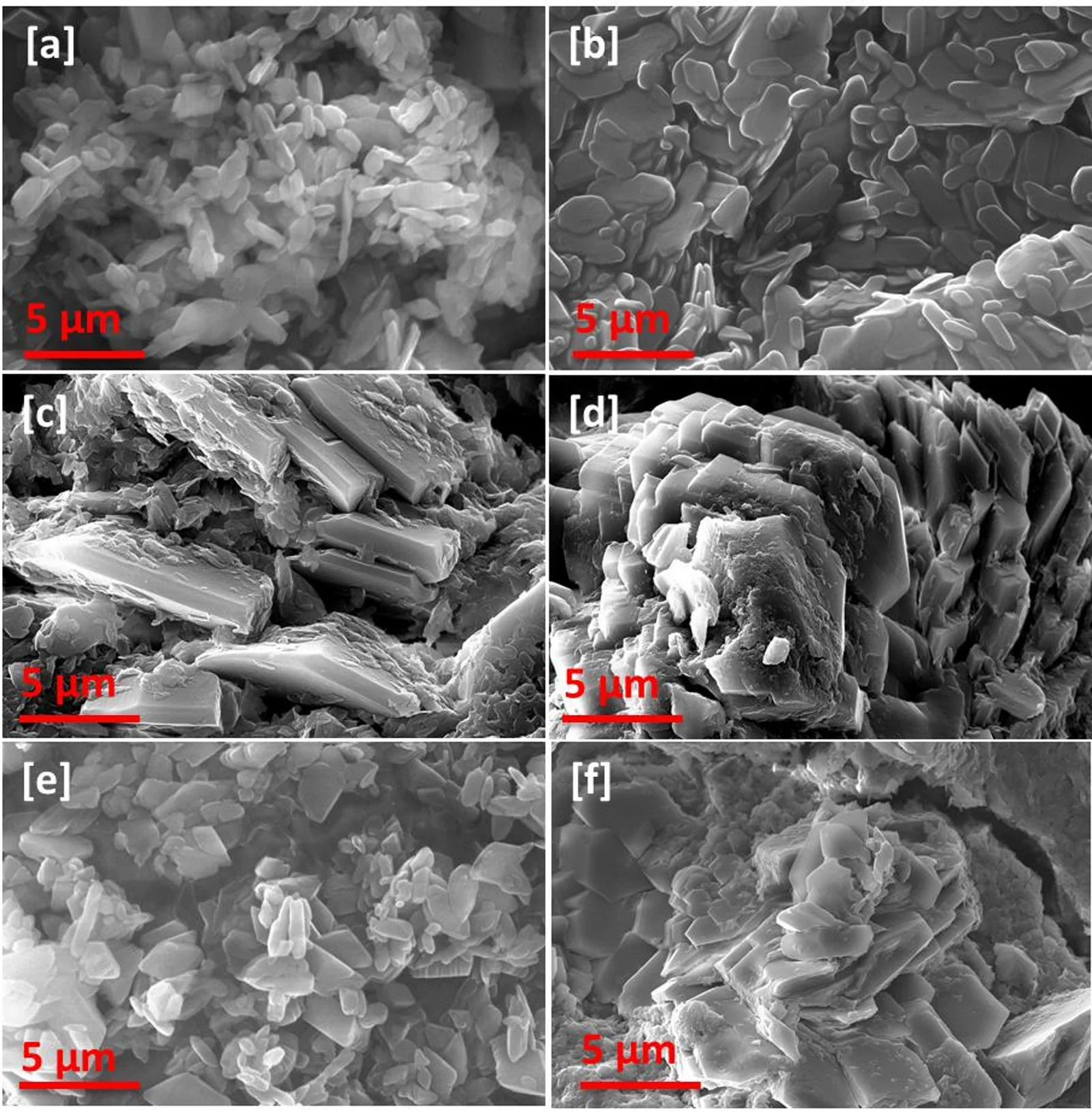
SEM of [**a**] Ca-BDC-NH_2_, [**b**] Zn-BDC-NH_2_, [**c**] Ca-Zn-BDC-NH_2_, and its chelating with chitosan [**d**] Ca-BDC-N = Chs, [**e**] Zn-BDC-N = Chs, and [**f**] Ca-Zn-BDC-N = Chs

Scanning Electron Microscopy (SEM) is essential for visualizing the morphology, particle size, and successful modification of MOFs (Fig. 2). The SEM images of pure Zn-MOF typically reveal a highly ordered, crystalline nature. It appears as octahedral micro-crystals with well-defined, sharp edges and smooth facets. The surface of the crystals was cleaned and free of amorphous debris. The crystals typically range from 500 nm to 5 μm. The introduction of calcium as a second metal node changed the crystal growth morphology; it was observed as rod-like structures. The surface was slightly “rougher” than the pure Zn-MOFs due to the competitive coordination between Zn and Ca nodes during nucleation. The SEM of the modified product provides the most visual proof of composite formation. The sharp edges of the MOF crystals appeared blurred. SEM showed the MOF crystals embedded within the cross-linked network of the chitosan. The distribution showed homogeneous particle shape.

**Fig. 3:**
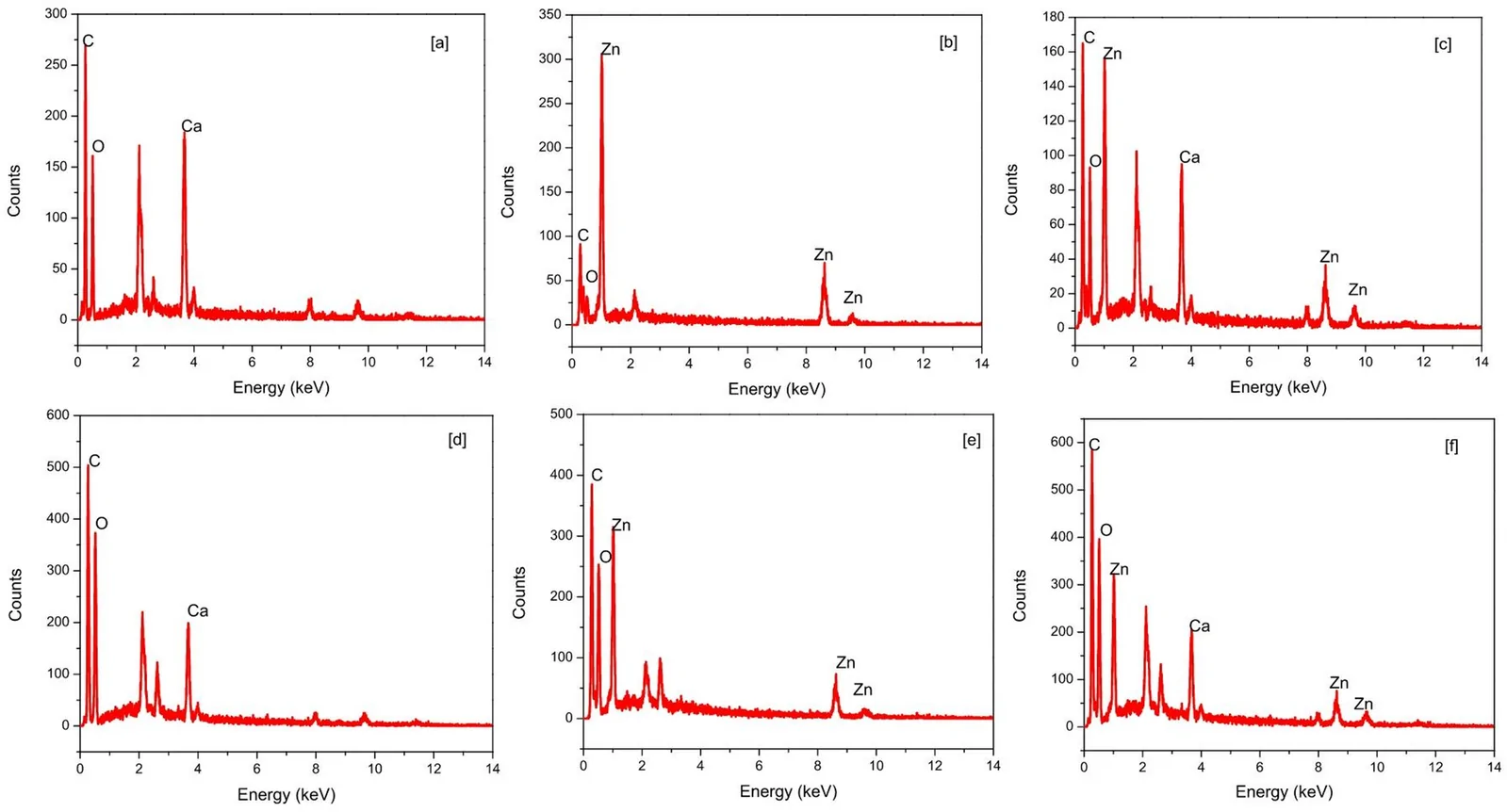
EDX of [**a**] Ca-BDC-NH_2_, [**b**] Zn-BDC-NH_2_, [**c**] Ca-Zn-BDC-NH_2_, and its chelating with chitosan [**d**] Ca-BDC-N= Chs, [**e**] Zn-BDC-N=Chs, and [**f**] Ca-Zn-BDC-N= Chs

Energy-dispersive X-ray spectroscopy (EDX) was employed to determine the surface elemental composition, verify the successful incorporation of metal nodes, and confirm the post-synthetic modification steps across the synthesized series (Fig. 3). The pristine MOFs exhibit sharp signals corresponding to their respective metal centers (Ca or Zn), heavily coordinated with C and O, and a distinct N signal stemming exclusively from the amino group of the 2-aminoterephthalate linker. Upon post-synthetic linkage with chitosan to form Ca-BDC-N=Chs and Zn-BDC-N=Chs, a systematic shift in the elemental ratios is observed. The C and O weight percentages increase significantly due to the carbon- and oxygen-rich backbone of the incoming biopolymer. The atomic ratio of N relative to the metal center increases, reflecting the combined nitrogen contribution from both the original amino-linker and the glucosamine units of chitosan. The ultimate multi-component platform contains all targeted elements simultaneously: C, O, N, Ca, and Zn.

**Fig. 4:**
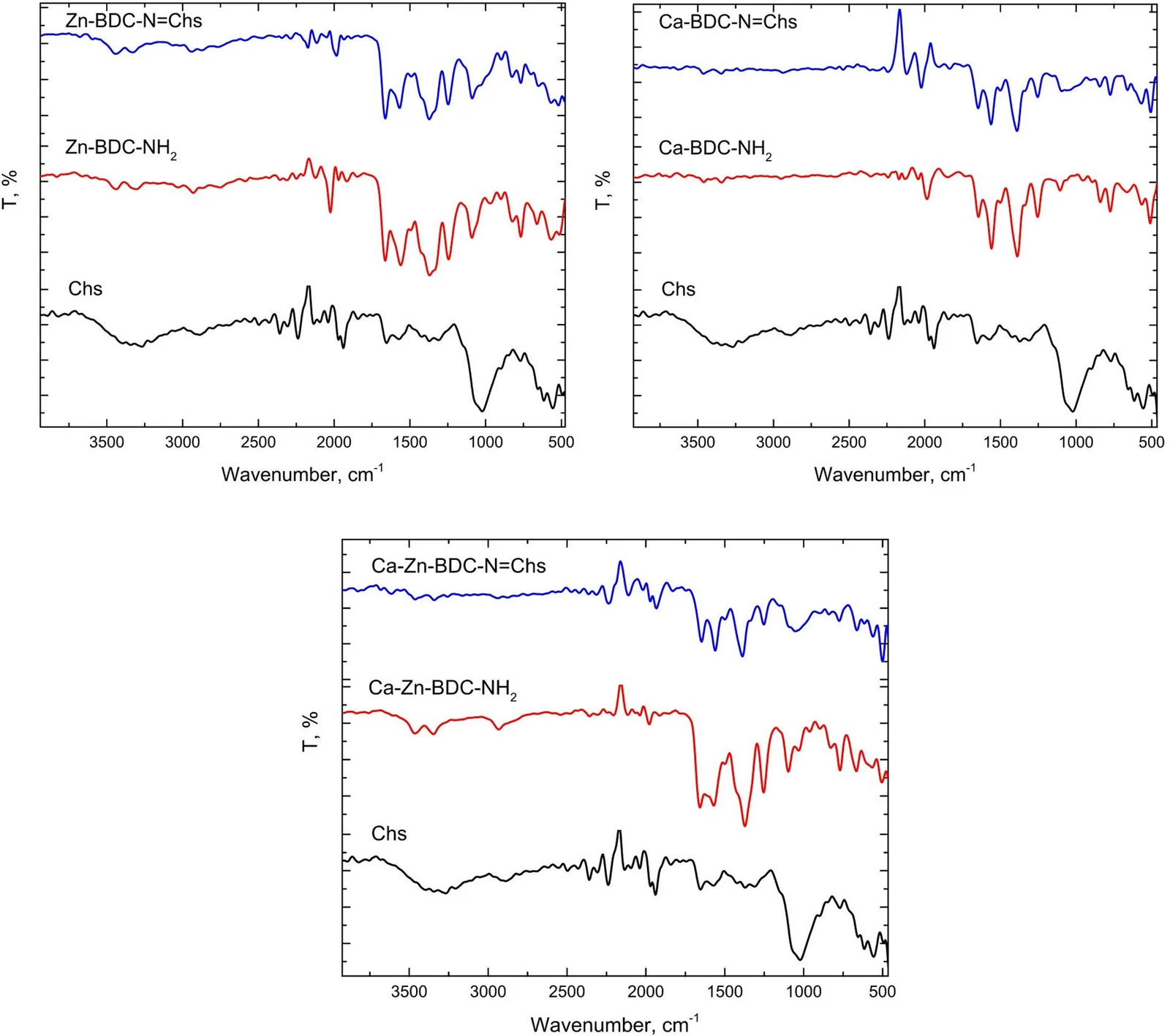
FTIR of Ca-BDC-NH_2_, Zn-BDC-NH_2_, Ca-Zn-BDC-NH_2_, and its chelating with chitosan

The FTIR spectrum of a Ca-BDC-NH_2_ (calcium 2-aminoterephthalate) MOF is a vital fingerprint that confirms the successful coordination of the calcium nodes with the amino-functionalized organic linker, as shown in Fig. 4. The interpretation is typically divided into three main regions: the amino/hydroxyl region, the carboxylate coordination region, and the aromatic fingerprint region. The spectrum showed peaks at 3200–3600 cm^-1^, these peaks confirm the presence of the pendant amine groups from the 2-aminoterephthalic acid linker. Also, peaks at 1550–1580 cm^-1^appeared; this confirmed successful MOF synthesis, because the sharp peak of the “free” carboxylic acid is usually found at 1680–1700 cm^-1^, but in our spectrum it disappeared and was replaced by two carboxylate salt peaks at 1550–1580 cm^-1^ for asymmetric carboxylate stretch and symmetric carboxylate stretch at 1380–1430. The FTIR spectrum of Zn-BDC-NH_2_ (zinc 2-aminoterephthalate) is shown in Fig. 4. The disappearance of the free carboxylic acid peak proves success of MOF formation. It is replaced by asymmetric carboxylate stretch at 1570–1600 cm^-1^ and symmetric carboxylate stretch at 1370–1430 cm^-1^. C-N stretching showed a band at 1250–1330 cm^-1^ confirming the bond between the aromatic ring and the amino group. Aromatic C = C was found at 1490 cm^-1^ and 1620 cm^-1^. C-H out-of-plane bending showed peaks around 760–800 cm^-1^, and when chitosan was modified with MOF, overlapping broad peaks at 3300–3500 cm^-1^ appeared. Because of the overlapping -OH and –NH_2_groups of the chitosan polymer. New peaks around 1650 cm^-1^ (Amide I) and 1590 cm^-1^ (Amide II) appeared; this is a strong interaction between the chitosan and the MOF surface.

**Fig. 5:**
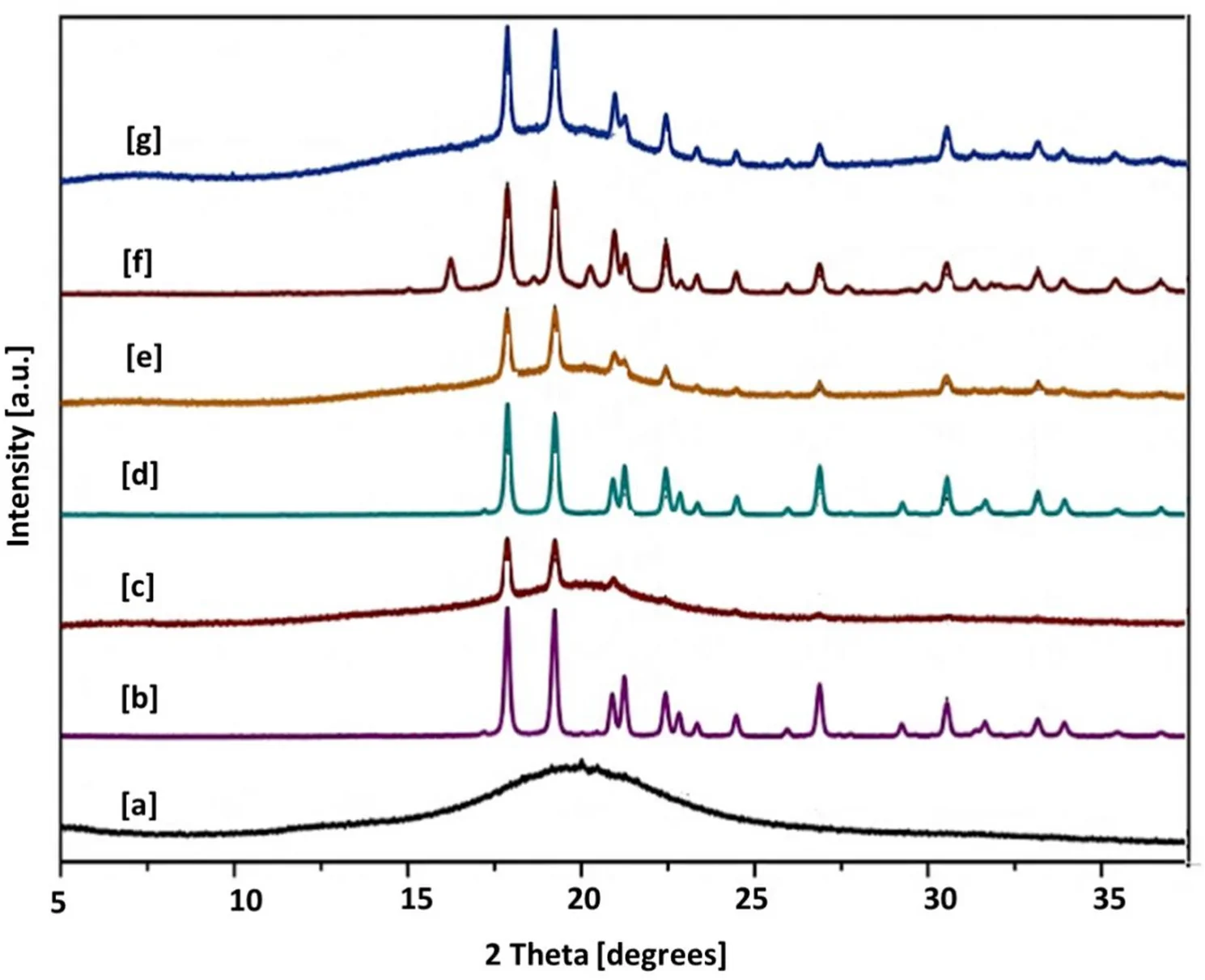
Stacked powder X-ray diffraction (PXRD) patterns of [**a**] pure chitosan (Chs), [**b**] pristine monometallic frameworks (Ca-BDC-NH_2_), [**c**] pristine monometallic frameworks (Zn-BDC-NH_2_), [**d**] post-synthetically modified monometallic composites (Ca-BDC-N = Chs), [**e**] post-synthetically modified monometallic composites (Zn-BDC-N=Chs), [**f**] pristine bimetallic framework (Ca-Zn-BDC-NH_2_), and [**g**] the final integrated bimetallic composite (Ca-Zn-BDC-N=Chs)

The crystalline structure, phase purity, and successful post-synthetic modification (PSM) of the prepared materials were evaluated using PXRD analysis. The stacked diffraction patterns are illustrated in Fig. 5. Chitosan (Chs) displays a single, broad, characteristic amorphous halo centered near 2 theta 20º, confirming its inherently semi-crystalline to amorphous macromolecular nature. In sharp contrast, the synthesized parent frameworks, Ca-BDC-NH_2_ and Zn-BDC-NH_2_ exhibit highly resolved, intense Bragg reflections, indicative of long-range order and high crystallinity. Ultimately, the final multi-component platform, Ca-Zn-BDC-N=Chs, successfully merges all structural features. It preserves the highly defined, intense bimetallic reflections alongside the underlying amorphous contribution from the covalently bound chitosan layer.

### Growth characteristics

A plant that is sensitive to excessive salt is quinoa. Plant biomass, germination time, and germination rate were all adversely affected by high salinity [31]. Two genotypes of quinoa, 1RE and 3YE, were cultivated in Ras Sudr, Egypt, which has a high salinity of more than 9600 parts per million. Twenty parts per million of the following compounds were sprayed on quinoa seedlings 25 and 50 days after planting: Ca-BDC-NH_2_, Zn-BDC-NH_2_, Chs, Ca-BDC-N=Chs, Zn-BDC-N=Chs, Ca-Zn-BDC-NH_2_, and Ca-Zn-BDC-N=Chs. Ten days following the second spray, vegetative samples were collected to quantify quinoa’s chemical, biological, and physiological characteristics as well as some growth and yield indices.

**Fig. 6:**
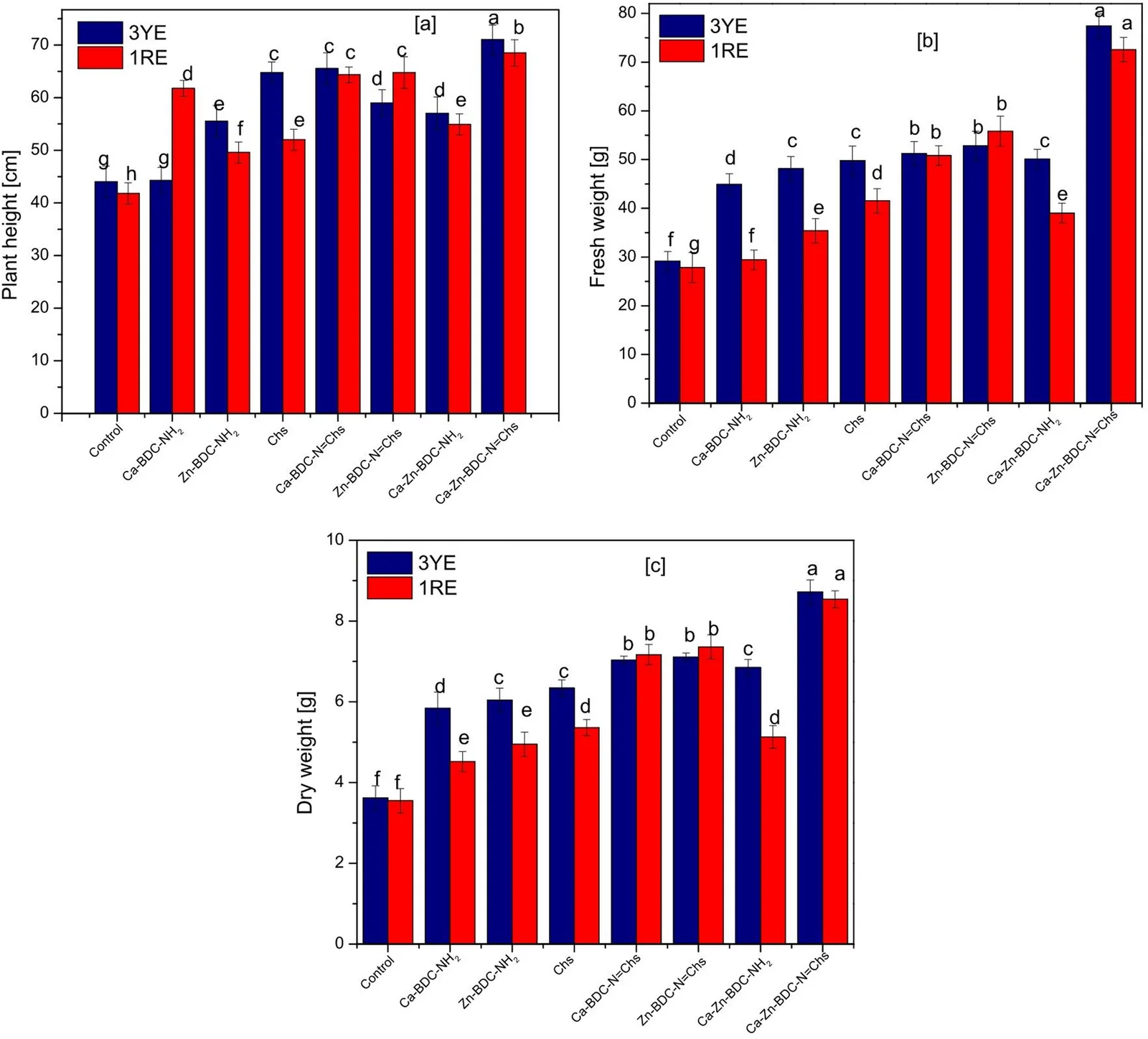
Effect of chitosan-modified mono-metallic and di-metallic frameworks on the vegetative growth of quinoa. (**a**) Average plant length (cm), (**b**) fresh weight (**g**), and (**c**) dry weight (**g**) measured at 10 days post-treatment. Error bars represent the standard deviation (SD) of triplicate measurements. Different letters above the bars indicate significant differences according to Duncan’s Multiple Range Test (*p* < 0.05)

After 60 days of planting, the results in Table 1; Fig. 6 demonstrate that the plant lengths increased by 62.57%, 30.43%, 44.23%, 51.34%, 36.07%, 22.44%, and 23.56%, respectively, following the application of treatments with Ca-Zn-BDC-N=Chs, Ca-Zn-BDC-NH_2_, Zn-BDC-N=Chs, Ca-BDC-N=Chs, Chs, Zn-BDC-NH_2_, and Ca-BDC-NH_2_ in comparison to the control. There were no discernible differences between the genotypes when compared. The results indicated that the Ca-Zn-BDC-N = Chs spray treatment produced the highest rates of length for both quinoa genotypes when the genotypes and spray treatments interacted. Spraying with Ca-Zn-BDC-N=Chs, Ca-Zn-BDC-NH_2_, Zn-BDC-N=Chs, Ca-BDC-N=Chs, Chs, Zn-BDC-NH_2_, and Ca-BDC-NH_2_ increased fresh weight by 2.63, 1.56, 1.91, 1.79, 1.6, 1.47, and 1.30 times, respectively, in the same situation. Additionally, when quinoa seedlings were externally sprayed with Ca-Zn-BDC-N=Chs, Ca-Zn-BDC-NH_2_, Zn-BDC-N=Chs, Ca-BDC-N=Chs, Chs, Zn-BDC-NH_2_, and Ca-BDC-NH_2_, respectively, their dry weight increased 2.41, 1.67, 2.02, 1.98, 1.63, 1.53, and 1.45 times in comparison to plants under salt stress. In terms of the difference between the two genetic compositions, the 3YE genotype fared better in fresh and dry weight than the 1RE genotype. Likewise, when paired with the genetic composition and external spray treatments, the Ca-Zn-BDC-N=Chs treatment yielded the highest fresh and dry weight values with two quinoa genotypes.

**Table 1:** Effect of external spray treatments on vegetative growth characteristics of two quinoa genotypes (3YE and 1RE) under salt stress conditions

External applications	Growth characteristics								
	Plant height (cm plant^-1^)			Fresh weight (g plant^-1^)			Dry weight (g plant^-1^)		
	3YE	1RE	Mean	3YE	1RE	Mean	3YE	1RE	Mean
Control	44.01 g	41.82 h	**42.91 F**	29.15 f	27.83 g	**28.49 F**	3.62 f	3.55 f	**3.58 E**
Ca-BDC-NH_2_	44.26 g	61.77 d	**53.02 E**	44.91 d	29.44 f	**37.17 E**	5.84 d	4.52 e	**5.18 D**
Zn- BDC-NH_2_	55.51 e	49.58 f	**52.54 E**	48.12 c	35.40 e	**41.76 D**	6.04 c	4.95 e	**5.49 D**
Chs	64.77 c	52.01 e	**58.39 D**	49.78 c	41.54 d	**45.66 C**	6.34 c	5.36 d	**5.85 C**
Ca-BDC-N=Chs	65.54 c	64.34 c	**64.94 B**	51.21 b	50.82 b	**51.01 B**	7.03 b	7.17 b	**7.10 B**
Zn-BDC-N=Chs	59.01 d	64.77 c	**61.89 C**	52.80 b	55.81 b	**54.30 B**	7.11 b	7.36 b	**7.23 B**
Ca-Zn-BDC-NH_2_	57.03 d	54.91 e	**55.97 D**	50.11 c	39.03 e	**44.57 C**	6.85 c	5.13 d	**5.99 C**
Ca-Zn-BDC-N=Chs	71.02 a	68.51 b	**69.76 A**	77.41 a	72.56 a	**74.99 A**	8.72 a	8.54 a	**8.63 A**
Mean	**57.64 A**	**57.21 A**		**50.44 A**	**44.05 B**		**6.44 A**	**5.82 B**	

Values followed by the same letter in columns are not different at *p* < 0.05 by Duncan’s multiple range tests

*Chs* Chitosan

### Biological yield

**Fig. 7:**
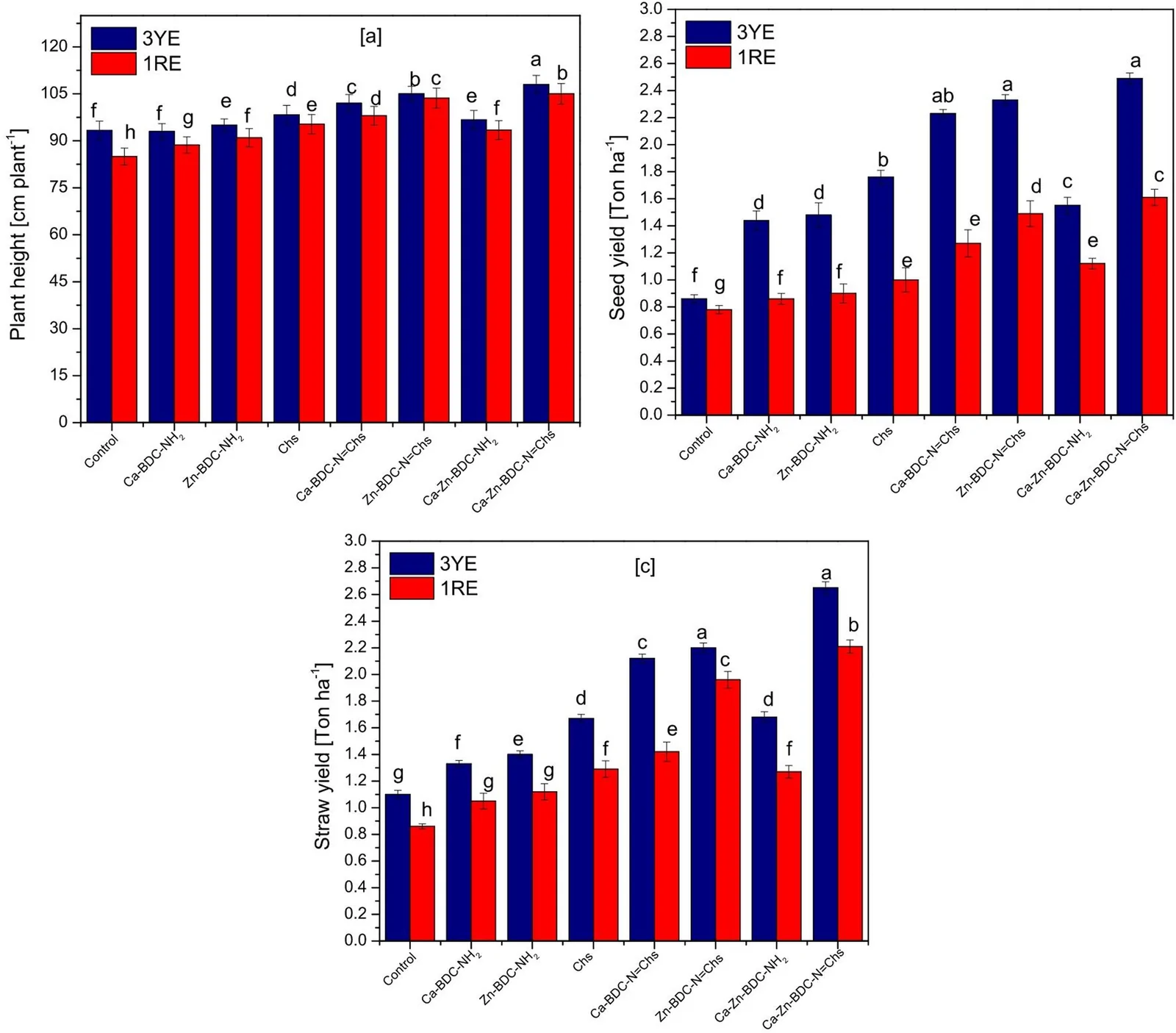
Evaluation of (**a**) Plant height (cm), (**b**) Seed yield (Ton ha^− 1^), and (**c**) Straw yield (Ton ha^− 1^) of quinoa under various treatments with chitosan-modified mono-metallic and di-metallic frameworks. Error bars represent the standard deviation (SD) of triplicate measurements. Different letters above the bars indicate significant differences according to Duncan’s Multiple Range Test (*p* < 0.05)

Quinoa crops are adversely affected by abiotic stress, such as salt stress, which results in inflorescence drop, grain shrinkage, decreased yield, decreased thousand-grain weight, and poor grain quality [31]. Quinoa seed yield, straw, and plant height at maturity, and harvest are all impacted by exogenous spraying with the Ca-Zn-BDC-N=Chs, Ca-Zn-BDC-NH_2_, Zn-BDC-N=Chs, Ca-BDC-N=Chs, Chs, Zn-BDC-NH_2_, and Ca-BDC-NH_2_, as indicated by the data in Table 2; Fig. 7. In comparison to the salt-stressed control, the results in the table indicate that outdoor spraying with the Ca-Zn-BDC-N=Chs, Ca-Zn-BDC-NH_2_, Zn-BDC-N=Chs, Ca-BDC-N=Chs, and Chs at harvest increased plant height by 19.45%, 6.62%, 17.01%, 12.16%, and 8.60%, respectively. The increase in quinoa plant height at harvest, however, did not differ significantly between the Ca-BDC-NH_2_ and Zn-BDC-NH_2_ spraying treatments. The 3YE variety fared better than the 1RE genotype in terms of increasing plant length at harvest, according to the results of a comparison of the genotypes under study. Accordingly, the Ca-Zn-BDC-N=Chs spray treatment was the most successful in extending plant length for both quinoa genotypes under saline circumstances, according to the results of the interaction between external spray treatments and genotypes. Grain and straw yields have clearly improved, according to the statistics in the same table and figure. In comparison to plants under salinity, quinoa seedlings under salinity exhibited a 2.50, 1.62, 2.33, 2.13, 1.68, 1.45, and 1.40 times increase in grain yield and a 2.48, 1.50, 2.12, 1.81, 1.51, 1.28, and 1.21 times increase in straw yield when external spray treatments of Ca-Zn-BDC-N=Chs, Ca-Zn-BDC-NH_2_, Zn-BDC-N = Chs, Ca-BDC-N=Chs, Chs, Zn-BDC-NH_2_, and Ca-BDC-NH_2_ were applied, respectively. When the genotypes were evaluated, the 3YE genotype was able to significantly increase grain and straw yield in contrast to the other genotypes. The Ca-Zn-BDC-N=Chs spray treatment achieved the highest mean value of increase in grain and straw production for the two quinoa genetic compositions when the interaction between genetic compositions and spray treatments was compared.

**Table 2:** Effect of external spray treatments on the biological yield of two quinoa genotypes (3YE and 1RE) under salt stress conditions

External applications	Biological yield								
	Plant height (cm plant^− 1^)			Seed yield (Ton ha^− 1^)			Straw yield (Ton ha^− 1^)		
	3YE	1RE	Mean	3YE	1RE	Mean	3YE	1RE	Mean
Control	93.33 f	85.00 h	**89.16 F**	0.86 f	0.78 g	**0.82 F**	1.10 g	0.86 h	**0.98 F**
Ca-BDC-NH_2_	93.00 f	88.67 g	**90.83 E**	1.44 d	0.86 f	**1.15 E**	1.33 f	1.05 g	**1.19 E**
Zn- BDC-NH_2_	95.00 e	91.00 f	**93.00 D**	1.48 d	0.90 f	**1.19 E**	1.40 e	1.12 g	**1.26 E**
Chs	98.33 d	95.33 e	**96.83 C**	1.76 b	1.00 e	**1.38 D**	1.67 d	1.29 f	**1.48 D**
Ca-BDC-N=Chs	102.00 c	98.00 d	**100.00 B**	2.23 ab	1.27 e	**1.75 C**	2.12 c	1.42 e	**1.77 C**
Zn-BDC-N=Chs	105.00 b	103.67 c	**104.33 AB**	2.33 a	1.49 d	**1.91 B**	2.20 b	1.96 c	**2.08 B**
Ca-Zn-BDC-NH_2_	96.72 e	93.41 f	**95.06 C**	1.55 c	1.12 e	**1.33 D**	1.68 d	1.27 f	**1.47 D**
Ca-Zn-BDC-N=Chs	108.00 a	105.00 b	**106.50 A**	2.49 a	1.61 c	**2.05 A**	2.65 a	2.21 b	**2.43 A**
Mean	**98.92 A**	**95.01 B**		**1.77 A**	**1.13 B**		**1.77 A**	**1.40 B**	

Values followed by the same letter in columns are not different at *p* < 0.05 by Duncan’s multiple range tests

*Chs* Chitosan

### Biochemical indices

#### Photosynthetic pigments

**Fig. 8:**
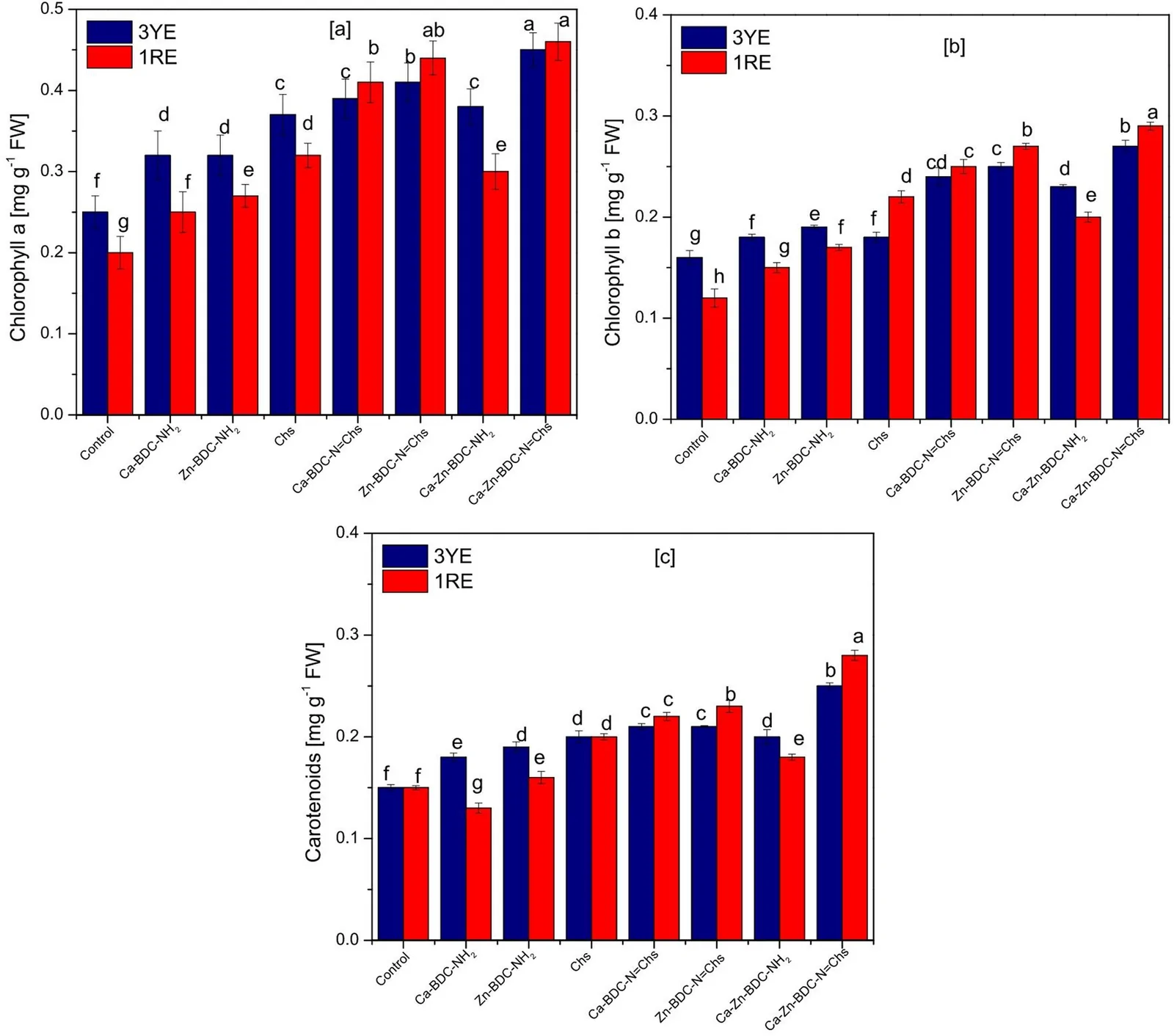
Comparative analysis of photosynthetic pigment concentrations in quinoa leaves across different treatments. (**a**) Chlorophyll *a*, (**b**) Chlorophyll *b*, and (**c**) Total carotenoid content (mg g^− 1^ FW). Error bars represent the standard deviation of the mean. Statistical significance (*p* < 0.05) is denoted by different alphabetical letters above the bars

Chlorophyll *a* and *b* concentrations as well as carotenoids have increased, and photosynthetic pigments have improved, according to the data referenced in Table 3; Fig. 8. When Ca-Zn-BDC-N=Chs, Ca-Zn-BDC-NH_2_, Zn-BDC-N=Chs, Ca-BDC-N=Chs, Chs, and Zn-BDC-NH_2_ were sprayed on quinoa seedlings, the percentage of chlorophyll *a* rose 2.04, 1.54, 1.91, 1.82, 1.54, and 1.32 times higher than the control, respectively. After applying Ca-Zn-BDC-N=Chs, Ca-Zn-BDC-NH_2_, Zn-BDC-N=Chs, Ca-BDC-N=Chs, Chs, and Zn-BDC-NH_2_ sprays, the percentage of chlorophyll *b* rose by 2.00, 1.50, 1.86, 1.71, 1.43, and 1.28 times in comparison to the control, respectively. Following external spraying with Ca-Zn-BDC-N=Chs, Ca-Zn-BDC-NH_2_, Zn-BDC-N=Chs, Ca-BDC-N=Chs, Chs, and Zn-BDC-NH_2_ treatments, the amount of carotenoids rose 1.73, 1.27, 1.47, 1.4, 1.33, and 1.13 times in comparison to the control, respectively. When compared to plants under salt stress, the Ca-BDC-NH_2_ spraying treatment did not significantly alter the amount of carotenoid pigments and chlorophyll *a* and *b*. The 3YE variety performed better than the other variety, 1RE, in terms of boosting carotenoids and chlorophyll *a*, according to a genotype comparison. The results of the investigation into the interaction between the genotypes and the spray treatments demonstrated that the two genotypes under investigation accumulated photosynthetic pigments, represented by chlorophyll *a* and *b*, and carotenoids at the maximum rate when spraying with Ca-Zn-BDC-N=Chs.

**Table 3:** Effects of external spray treatments on the photosynthetic pigments of two quinoa genotypes (3YE and 1RE) under salt stress conditions

External applications	Photosynthetic pigments (mg g^− 1^ FW)								
	Chlorophyll (a)			Chlorophyll (b)			Carotenoid		
	3YE	1RE	Mean	3YE	1RE	Mean	3YE	1RE	Mean
Control	0.25 f	0.20 g	**0.22 E**	0.16 g	0.12 h	**0.14 G**	0.15 f	0.15 f	**0.15 F**
Ca-BDC-NH_2_	0.32 d	0.25 f	**0.28 D**	0.18 f	0.15 g	**0.16 F**	0.18 e	0.13 g	**0.15 F**
Zn-BDC-NH_2_	0.32 d	0.27 e	**0.29 D**	0.19 e	0.17 f	**0.18 E**	0.19 d	0.16 e	**0.17 E**
Chs	0.37 c	0.32 d	**0.34 C**	0.18 f	0.22 d	**0.20 D**	0.20 d	0.20 d	**0.20 C**
Ca-BDC-N=Chs	0.39 c	0.41 b	**0.40 B**	0.24 cd	0.25 c	**0.24 C**	0.21 c	0.22 c	**0.21 BC**
Zn-BDC-N=Chs	0.41 b	0.44 ab	**0.42 B**	0.25 c	0.27 b	**0.26 B**	0.21 c	0.23 b	**0.22 B**
Ca-Zn-BDC-NH_2_	0.38 c	0.30 e	**0.34 C**	0.23 d	0.20 e	**0.21 D**	0.20 d	0.18 e	**0.19 D**
Ca-Zn-BDC-N=Chs	0.45 a	0.46 a	**0.45 A**	0.27 b	0.29 a	**0.28 A**	0.25 b	0.28 a	**0.26 A**
Mean	**0.36 A**	**0.33 B**		**0.21 A**	**0.21 A**		**0.20 A**	**0.19 B**	

Values followed by the same letter in columns are not different at *p* < 0.05 by Duncan’s multiple range tests

*Chs* Chitosan

#### Stress physiological indices

Plant cells under salt stress experience an increase in free radical accumulation, which causes an imbalance in osmotic balance and the oxidation of cell membrane lipids, resulting in the loss of selective permeability of plasma membranes.

##### Malondialdehyde (MDA)

**Fig. 9:**
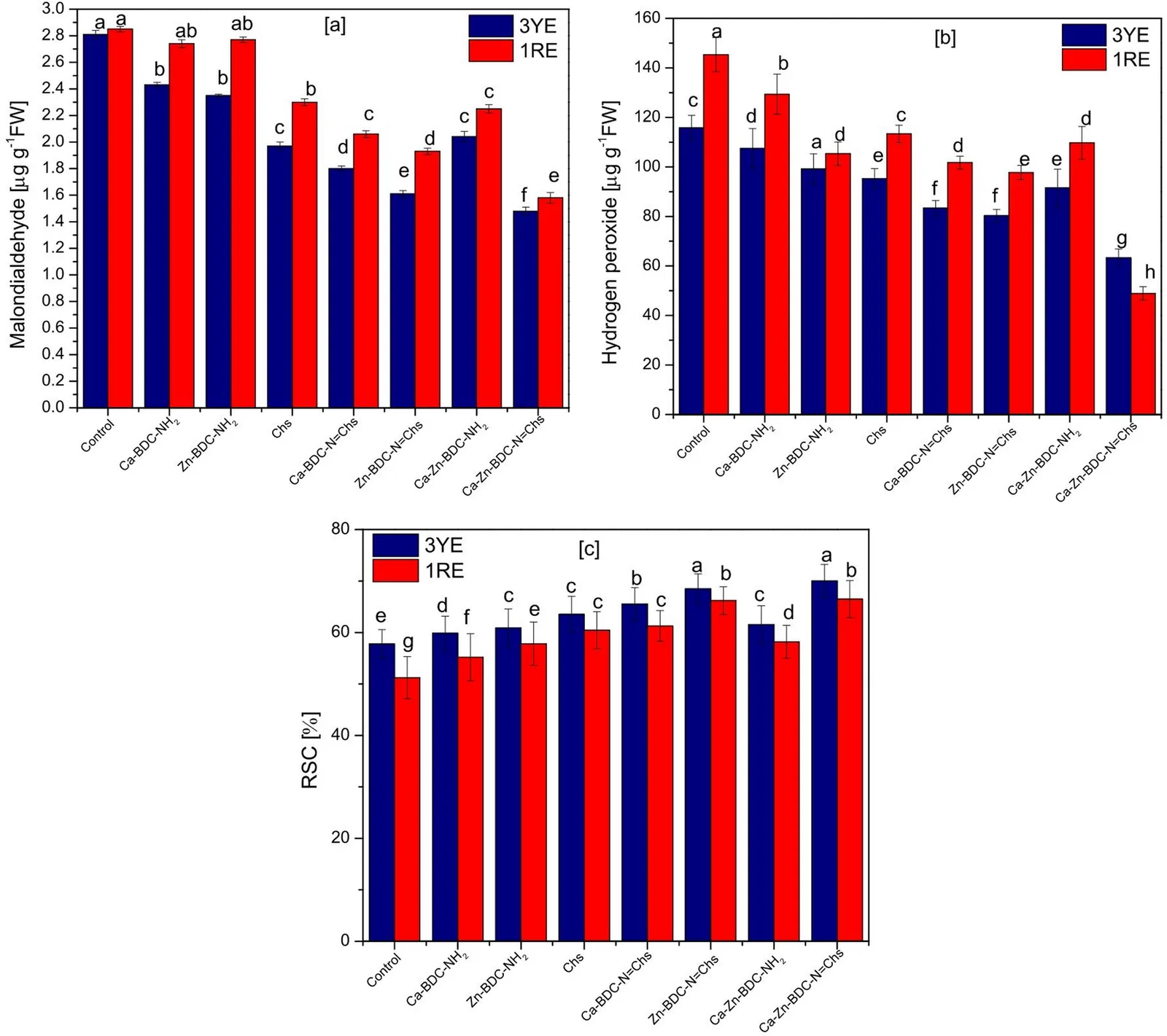
Assessment of oxidative stress markers and antioxidant capacity in quinoa tissues under various treatments. (**a**) Malondialdehyde (MDA) content (µg g^− 1^ FW), (**b**) Hydrogen peroxide accumulation (µg g^− 1^ FW), and (**c**) Percent of radical scavenging capacity (RSC%). Data are presented as mean SD (*n* = 3). Different letters indicate significant differences at *p* < 0.05 according to Duncan’s Multiple Range Test

The information shown in Table 4 and Fig. 9 suggests that external spray treatments have reduced the buildup of MDA. The Ca-Zn-BDC-N=Chs spray treatment was the most effective in lowering the MDA content in the leaves of quinoa seedlings, resulting in a 45.94% reduction. Zn-BDC-N=Chs, Ca-BDC-N=Chs, Chs and Ca-Zn-BDC-NH_2_followed with decreases of 37.45%, 31.80%, 24.73%, and 24.38%, respectively. With decreases of 8.83% and 9.54%, respectively, the Ca-BDC-NH_2_ and Zn-BDC-NH_2_spray treatments were the ones that decreased MDA concentrations. In the presence of salt stress, the 3YE genotype was able to reduce MDA more effectively than the 1RE genotype. The findings of examining the interaction between the genotypes and spray treatments demonstrated that, under saline field conditions, the Ca-Zn-BDC-N=Chs spray treatment outperformed both genotypes in lowering MDA content.

**Table 4:** Effect of external spray treatments on stress physiological indices (MDA, H_2_O_2_, and RSC %) of two quinoa genotypes (3YE and 1RE) grown under salt stress conditions

External applications	Stress physiological indices								
	Malondialdehyde (µg g^− 1^ FW)			Hydrogen peroxide (µg g^− 1^ FW)			RSC (%)		
	3YE	1RE	Mean	3YE	1RE	Mean	3YE	1RE	Mean
Control	2.81 a	2.85 a	**2.83 A**	115.84 c	145.34 a	**130.59 A**	57.76 e	51.22 g	**54.49 E**
Ca-BDC-NH_2_	2.43 b	2.74 ab	**2.58 B**	107.47 d	129.35 b	**118.41 B**	59.86 d	55.18 f	**57.52 D**
Zn-BDC-NH_2_	2.35 b	2.77 ab	**2.56 B**	99.26 e	105.34 d	**102.30 C**	60.89 c	57.82 e	**59.35 C**
Chs	1.97 c	2.30 b	**2.13 C**	95.33 e	113.35 c	**104.34 C**	63.53 c	60.44 c	**61.98 B**
Ca-BDC-N=Chs	1.80 d	2.06 c	**1.93 D**	83.43 f	101.74 d	**92.58 E**	65.51 b	61.26 c	**63.38 B**
Zn-BDC-N=Chs	1.61 e	1.93 d	**1.77 E**	80.33 f	97.73 e	**89.03 E**	68.48 a	66.21 b	**67.34 A**
Ca-Zn-BDC-NH_2_	2.04 c	2.25 c	**2.14 C**	91.51 e	109.71 d	**100.61 D**	61.52 c	58.19 d	**59.85 C**
Ca-Zn-BDC-N=Chs	1.48 f	1.58 e	**1.53 F**	63.31 g	48.94 h	**56.12 F**	70.03 a	66.48 b	**68.25 A**
Mean	**2.06 B**	**2.31 A**		**92.06 B**	**106.44 A**		**63.45 A**	**59.60 B**	

Values followed by the same letter in columns are not different at *p* < 0.05 by Duncan’s multiple range tests

*Chs* Chitosan, *RSC* Radical scavenging capacity

##### Hydrogen peroxide (H_2_O_2_)

The results of the same table and Fig. 9 demonstrated that the Ca-Zn-BDC-N=Chs, Zn-BDC-N=Chs, and Ca-BDC-N=Chs spraying treatments were the most effective in lowering the hydrogen peroxide concentration in quinoa leaf seedlings, resulting in reductions of 57.03%, 31.82%, and 29.11%, respectively. Following this, there was a decrease in H_2_O_2_ accumulation of 22.96%, 20.10%, 21.66%, and 9.33%, respectively, due to the spraying treatments with Ca-Zn-BDC-NH_2_, Chs, Zn-BDC-NH_2_, and Ca-BDC-NH_2_. Unlike the genotype 1RE, the genotype 3YE was able to decrease H_2_O_2_ buildup. The data tabulated in the same table, which examined the interaction between spray treatments and genetic compositions, demonstrated that the Ca-Zn-BDC-N=Chs spray treatment was able to demonstrate the highest rate of lowering the H_2_O_2_ concentration with the two genetic compositions of quinoa under saline cultivation conditions.

##### Percent of Radical Scavenging Capacity (RSC%)

RSC%, also known as radical inhibition percentage, measures an antioxidant’s ability to neutralize free radicals; it evaluates how effectively a substance prevents oxidative damage. The Ca-Zn-BDC-N = Chs spray treatment had the highest free radical scavenging rate in quinoa seedling leaves, according to the findings in Table 4; Fig. 9. Zn-BDC-N=Chs, Ca-BDC-N=Chs and Chs had rates at 67.34%, 63.38%, and 61.98% respectively. Additionally, Ca-BDC-NH_2,_ Ca-Zn-BDC-NH_2_ and Zn-BDC-NH_2_ showed lower rates at57.52%, 59.85 and 59.35%, respectively. Comparing the genotypes, it was found that the 3YE genotype outperformed the 1RE variety in terms of free radical scavenging rate. In comparison to the two quinoa genotypes, 3YE and 1RE, cultivated under salt stress, the Ca-Zn-BDC-N=Chs spray treatment demonstrated the highest free radical scavenging rate when genotypes and spray treatments were taken into account.

Based on previous reports on related coordination complexes [66, 67], the high antioxidant activity of Ca-Zn-BDC-N=Chs can be attributed to the unhindered availability of the free amine groups attached to the 2-aminoterephthalic linker and chitosan molecule. Free primary amines are highly capable of donating hydrogen atoms or transferring electrons to stabilize free radicals. Additionally, the porous nature of the pure MOF ensures high surface area accessibility for the radical molecules to interact with these active sites. Across all evaluated material groups, a statistically significant reduction in RSC% is observed when transitioning from the 3YE condition to the 1RE condition.

#### Antioxidant enzymes

##### Superoxide Dismutase (SOD) isozymes

**Fig. 10:**
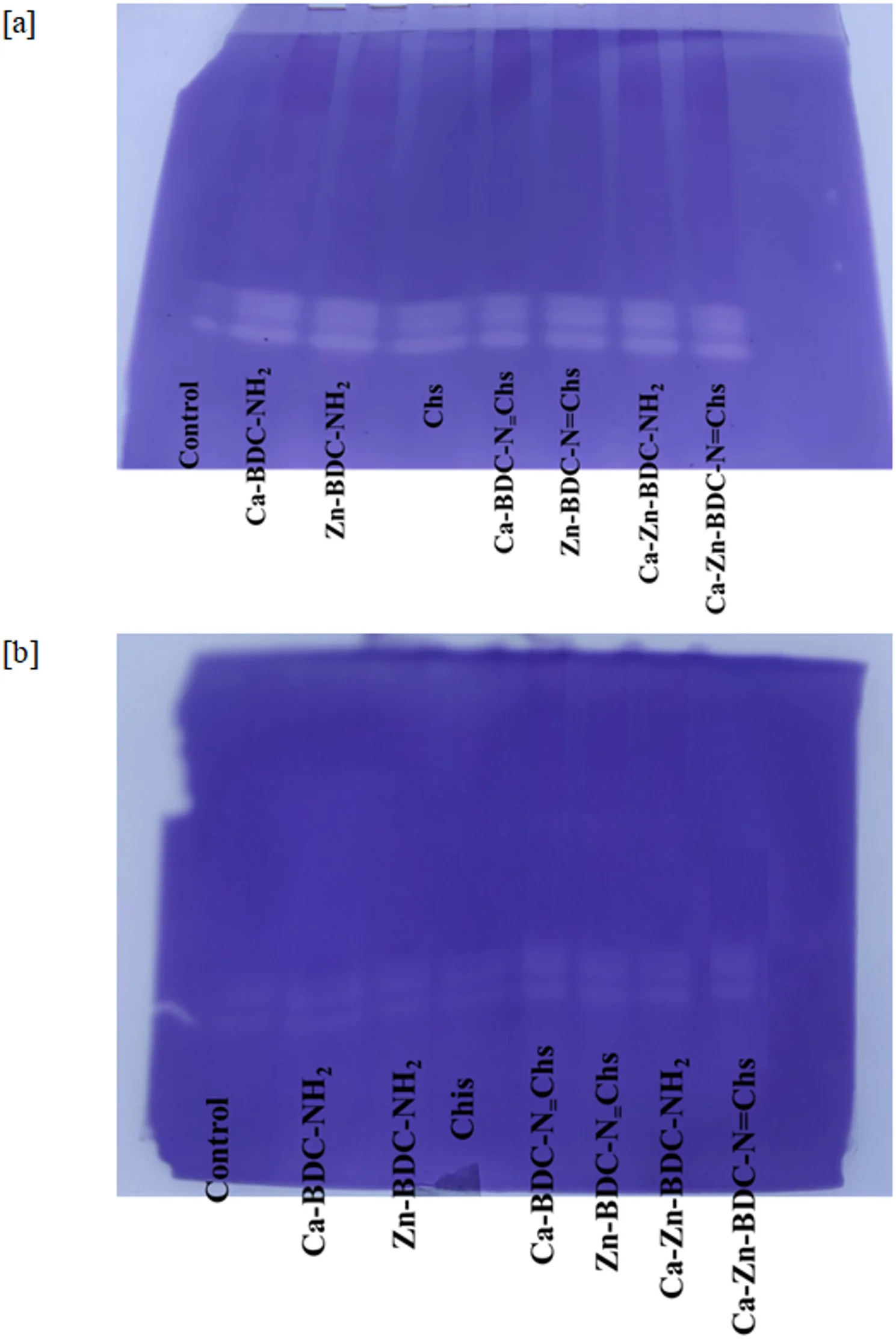
Native-PAGE zymogram and quantitative analysis of superoxide dismutase (SOD) isoenzymes in quinoa leaves [**a**] 3YE variety, [**b**]1RE variety

The first line of defense against ROS in a cell is the metalloenzyme known as the SOD pattern [68]. SOD patterns for the two quinoa genotypes (3YE and 1RE) in the current investigation showed roughly four bands, as indicated in Table 5; Fig. 10. External spray treatments of Chs and Ca-BDC-N=Chs caused the initial band on the green leaves of the 3YE genotype cultivated in saline conditions to vanish. In the 3YE genotype cultivated in saline circumstances, the density of the second band rose with all external spray treatments in comparison to the control. Except for the Ca-BDC-N=Chs and Zn-BDC-N=Chs treatments, all spray treatments reduced the third band’s density in the 3YE genotype. When comparing the 3YE genotype to plants under salt stress, the density of the fourth band increased with all spray treatments except Ca-BDC-N=Chs. All spray treatments, with the exception of the Ca-BDC-N=Chs treatment, enhanced the density of the first band in the 1RE genotype cultivated in saline conditions as compared to the control. In comparison to the control, the density of the second band increased with all spray treatments except Zn-BDC-NH_2_. In contrast to the control, the third band on the leaves of the genotype 1RE cultivated in saline conditions vanished after spray treatments with Ca-BDC-NH_2_, Chs, and Zn-BDC-N=Chs. When comparing the genotype 1RE leaves to the stressed plants, the density of the fourth band of the SOD enzyme decreased with all external spray treatments except Chs, and Zn-BDC-N=Chs.

**Table 5:** Effect of external spray treatments on isozymic pattern for superoxide dismutase of two quinoa genotypes (3YE and 1RE) grown under salt stress conditions

External applications	Band intensity									
	3YE				Total bands	1RE				Total bands
	Band 1Band 1	Band 2	Band 3	Band 4		Band 1	Band 2	Band 3	Band 4	
Control	1.00	2.16	1.66	2.68	**4**	1.49	1.81	1.58	2.87	**4**
Ca-BDC-NH_2_	1.14	2.52	1.46	3.00	**4**	2.09	3.43	**0**	2.22	**3**
Zn- BDC-NH_2_	1.78	2.25	1.26	2.99	**4**	3.17	1.00	2.56	2.70	**4**
Chs	**0**	2.49	1.39	2.95	**3**	1.91	3.18	**0**	2.90	**3**
Ca- BDC-N=Chs	**0**	2.18	1.68	2.28	**3**	1.38	1.84	2.86	2.43	**4**
Zn- BDC-N=Chs	2.19	2.67	1.75	3.02	**4**	1.83	3.86	**0**	3.40	**3**
Ca-Zn- BDC-NH_2_	1.21	2.01	1.69	2.31	**4**	1.87	2.35	**0**	2.67	**3**
Ca-Zn- BDC-N=Chs	1.32	2.38	1.55	2.94	**4**	1.81	1.97	2.37	2.40	**4**

Where 0: reference to no band, 1.00: reference to lowest band intensity, 3.02: reference to highest band intensity for 3YE genotype, and 3.86: reference to highest band intensity for 1RE genotype

*Chs* Chitosan

##### Peroxidase enzyme activity

**Fig. 11:**
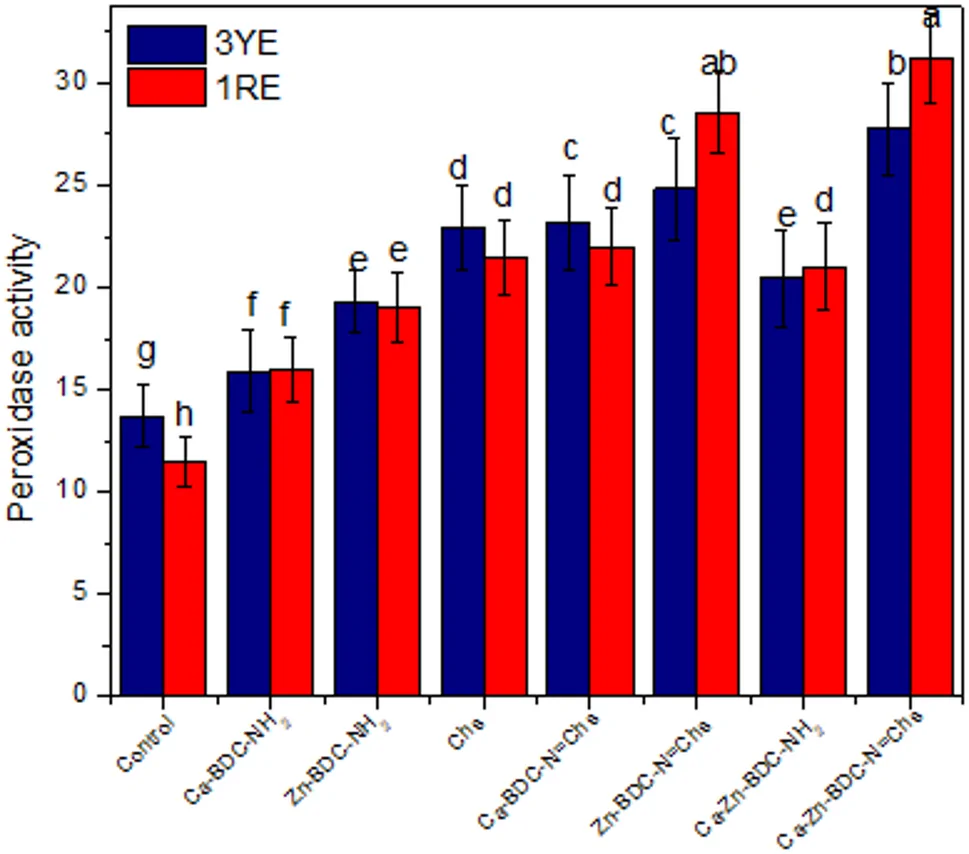
Evaluation of peroxidase (POD) enzyme activity (∆A460/3min/g FW) in quinoa tissues treated with different MOF-chitosan. Data represent the mean standard deviation (*n* = 3). Different letters indicate statistical significance (*p* < 0.05) according to Duncan’s Multiple Range Test

In comparison to plants under salt stress and not sprayed with any treatment, the data in Table 6, Fig. 11, demonstrate the effectiveness of spraying treatments with Ca-Zn-BDC-N=Chs, Ca-Zn-BDC-NH_2_, Zn-BDC-N=Chs, Ca-BDC-N=Chs, Chs, Zn-BDC-NH_2_, and Ca-BDC-NH_2_ in raising the activity of the peroxidase enzyme by 2.34, 1.65, 2.12, 1.79, 1.76, 1.52, and 1.27 times, respectively. The peroxidase enzyme’s activity was higher in the genotype 1RE than in the genotype 3YE when the genotypes were compared. In terms of how genotypes and spraying treatments interact, the Ca-Zn-BDC-N=Chs spraying treatment with the two quinoa genetic combinations (3YE and 1RE) outperformed the control for each of them in terms of raising the peroxidase enzyme’s activity.

**Table 6:** Effect of external spray treatments on peroxidase enzyme activity of two quinoa genotypes (3YE and 1RE) grown under salt stress conditions

External applications	Peroxidase activity (ΔAbs/ 3 min/g FW)		
	3YE	1RE	Mean
Control	13.73 g	11.46 h	**12.59 G**
Ca-BDC-NH_2_	15.92 f	15.97 f	**15.94 F**
Zn-BDC-NH_2_	19.32 e	19.06 e	**19.19 E**
Chs	22.92 d	21.49 d	**22.21 C**
Ca-BDC-N=Chs	23.21 c	21.98 d	**22.59 C**
Zn-BDC-N=Chs	24.83 c	28.57 ab	**26.70 B**
Ca-Zn-BDC-NH_2_	20.45 e	21.03 d	**20.74 D**
Ca-Zn-BDC-N=Chs	27.74 b	31.26 a	**29.50 A**
Mean	**21.01 B**	**21.35 A**	

Values followed by the same letter in columns are not different at *p* < 0.05 by Duncan’s multiple range tests

*Chs* Chitosan, *RSC* Radical scavenging capacity

#### Amino acid content in seed yield

**Fig. 12:**
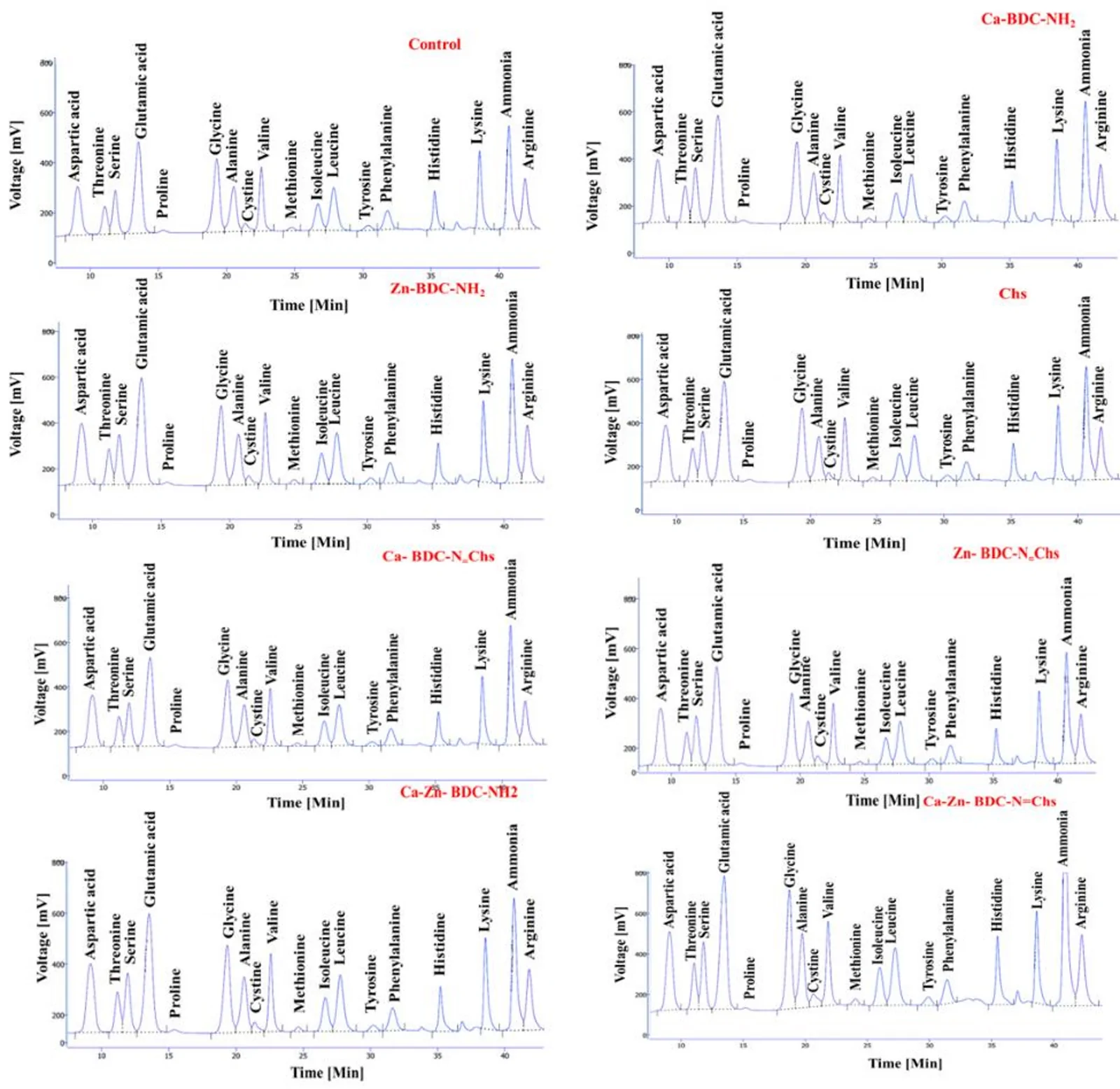
Comparative amino acid profiles for quinoa 3YE variety obtained via automated ion-exchange chromatography (Amino Acid Analyzer)

**Fig. 13:**
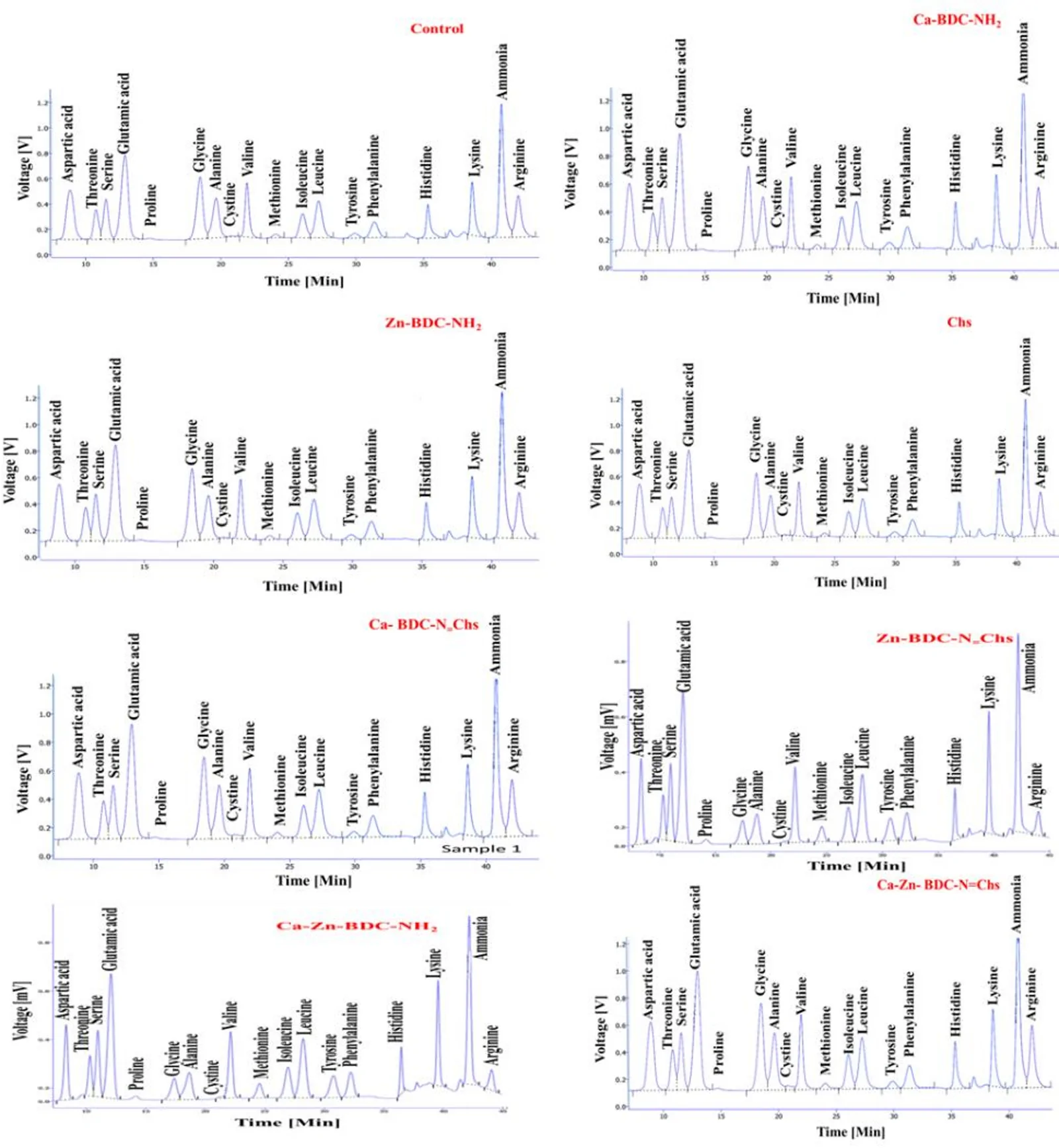
Amino acid analyzer chromatograms of quinoa 1RE variety under different treatment with mono, di-metallic MOF-modified chitosan

The amount and quality of amino acids in quinoa seeds cultivated in saline circumstances were examined in relation to the effects of spraying treatments with Ca-Zn-BDC-N=Chs, Ca-Zn-BDC-NH_2_, Zn-BDC-N=Chs, Ca-BDC-N=Chs, Chs, Zn-BDC-NH_2_, and Ca-BDC-NH_2_. Eight essential amino acids-threonine, valine, methionine, isoleucine, leucine, phenylalanine, histidine, and lysine-were found in quinoa seeds, according to the results in Table 7 and Figs. 12 and 13. The most prevalent amino acids in quinoa seeds were leucine and lysine. In quinoa seeds, methionine was the least prevalent essential amino acid. The seven non-essential amino acids aspartic acid, serine, glutamic acid, proline, glycine, alanine, and tyrosine are also present in quinoa seeds. The most prevalent amino acid was glutamic acid, which was followed by glycine and aspartic acid. The least prevalent amino acid in quinoa in saline circumstances, however, was tyrosine. Quinoa contains the semi-essential amino acids cystine and arginine. In comparison to the control, the amino acid content increased by 39.70%, 23.15%, 24.71%, 25.17%, 6.78% and 7.56% for the 1RE genotype and by 27.68%, 17.39%, 22.04%, 27.35%, 20.73% and 7.43% for the 3YE genotype after spraying treatments with Ca-Zn-BDC-N=Chs, Ca-Zn-BDC-NH_2_, Zn-BDC-N=Chs, Ca-BDC-N=Chs, Chs, and Ca-BDC-NH_2,_ respectively.

**Table 7:** Effect of external spray treatments on the amount of amino acids (mg g^− 1^) in the crushed seeds of two quinoa genotypes (3YE and 1RE) grown under salt stress conditions

Amino acids		External applications															
		Control		Ca-BDC-NH_2_		Zn-BDC-NH_2_		Chs		Ca-BDC-*N*=Chs		Zn-BDC-*N*=Chs		Ca-Zn-BDC-NH_2_		Ca-Zn-BDC-*N*=Chs	
		3YE	1RE	3YE	1RE	3YE	1RE	3YE	1RE	3YE	1RE	3YE	1RE	3YE	1RE	3YE	1RE
Essential	**Threonine**	3.49	4.72	4.24	5.11	4.15	4.77	4.76	4.56	5.07	5.51	4.83	5.53	4.13	5.13	4.93	6.25
	**Valine**	6.25	6.18	6.24	6.58	5.93	6.24	6.72	6.76	7.30	7.37	7.10	8.09	8.01	6.69	7.52	8.95
	**Methionine**	0.65	0.84	0.68	0.83	0.55	0.68	0.74	0.86	0.78	0.88	0.68	1.74	0.69	2.74	0.86	1.84
	**Isoleucine**	4.83	5.43	5.01	5.75	4.79	5.58	5.68	5.49	5.96	6.58	5.57	6.91	6.55	7.01	6.06	7.66
	**Leucine**	7.85	8.61	8.22	9.27	7.75	9.00	9.27	8.75	9.88	10.44	9.00	10.69	9.69	11.69	9.92	11.90
	**Phenylalanine**	4.22	5.14	4.34	5.42	4.13	5.31	4.86	4.84	5.10	6.44	4.90	6.62	3.96	5.65	5.07	7.45
	**Histidine**	4.73	5.78	4.84	5.78	4.77	5.30	5.28	7.70	5.87	7.05	5.37	6.59	5.02	6.00	5.78	7.16
	**Lysine**	7.38	7.68	7.37	8.53	7.03	8.18	8.13	8.43	8.64	9.54	8.18	9.18	8.38	10.02	8.42	10.56
	**Total**	**39.40**	**44.38**	**40.95**	**47.28**	**39.10**	**45.06**	**45.45**	**47.38**	**48.60**	**53.80**	**45.63**	**55.35**	**46.43**	**54.93**	**48.56**	**61.78**
Non- essential	**Aspartic acid**	8.17	12.81	10.67	13.81	10.40	13.89	11.97	11.96	12.68	16.10	11.70	15.91	10.79	16.70	12.82	17.29
	**Serine**	4.03	5.08	4.76	5.75	4.79	5.21	5.57	5.21	5.75	6.17	5.53	6.22	4.56	5.25	5.29	7.14
	**Glutamic acid**	18.34	22.10	20.56	24.37	20.40	23.15	23.98	22.36	24.74	28.67	23.79	27.56	24.00	29.56	24.61	30.54
	**Proline**	4.09	4.09	4.11	4.39	3.81	4.24	4.51	4.40	4.60	5.01	4.90	4.88	4.03	3.88	4.67	5.45
	**Glycine**	8.12	8.12	8.52	8.90	8.15	8.41	9.37	8.62	9.83	10.14	9.68	9.81	9.18	6.81	9.86	11.15
	**Alanine**	5.80	5.93	6.13	6.37	5.74	6.07	6.27	6.33	7.13	7.12	6.96	7.72	6.16	5.72	7.29	8.64
	**Tyrosine**	1.44	1.77	1.37	1.66	1.41	1.71	1.85	1.98	1.69	2.32	1.77	1.96	1.22	2.96	1.85	2.60
	**Total**	**50.00**	**59.90**	**56.12**	**65.26**	**54.70**	**62.68**	**63.53**	**60.86**	**66.41**	**75.54**	**64.33**	**74.06**	**59.94**	**70.88**	**66.37**	**82.80**
Semi- essential	**Cystine**	1.47	0.23	1.47	0.31	1.60	0.24	0.98	2.95	1.64	0.43	1.82	1.38	1.62	1.44	1.73	1.44
	**Arginine**	11.10	11.66	11.01	12.10	10.97	11.99	13.15	12.86	13.21	15.64	12.66	14.10	11.71	15.81	13.53	16.27
	**Total**	**12.57**	**11.89**	**12.48**	**12.41**	**12.57**	**12.23**	**14.13**	**15.81**	**14.85**	**16.07**	**14.48**	15.48	**13.33**	**17.25**	**15.27**	**17.71**
Total amino acids		**101.97**	**116.17**	**109.55**	**124.95**	**106.37**	**119.97**	**123.11**	**124.05**	**129.86**	**145.41**	**124.44**	**144.88**	**119.70**	**143.06**	**130.20**	**162.29**

The amino acids in the table were arranged (ascending) according to the retention time of amino acids, which were separated in a column of an amino acid analyzer apparatus

*Chs* Chitosan

The percentage of essential, semi-essential, and non-essential amino acids in crushed quinoa seeds cultivated in a saline environment is displayed in Table 8. The percentage of essential amino acids in the two genotypes under study decreased with each spray treatment as compared to the control. On the other hand, the percentage of non-essential amino acids in the two genotypes under study increased as a result of all spray treatments. However, the fraction of semi-essential amino acids in the 3YE genotype decreased with all spray treatments. In contrast to the control genotype 1RE, all spray treatments-aside from the Ca-BDC-NH_2_ and Zn-BDC-NH_2_ spray treatments-increased the percentage of semi-essential amino acids.

**Table 8:** Effect of external spray treatments on the percentage of essential, non-essential, and semi-essential amino acids in the crushed seeds of two quinoa genotypes (3YE and 1RE) grown under salt stress conditions

External applications	Essential amino acids (%)		Non-essential amino acids (%)		Semi-essential amino acids (%)	
	3YE	1RE	3YE	1RE	3YE	1RE
Control	38.64	38.20	49.04	51.56	12.32	10.24
Ca-BDC-NH_2_	37.38	37.84	51.22	52.23	11.39	9.93
Zn-BDC-NH_2_	36.76	37.56	51.43	52.25	11.81	10.20
Chs	36.92	38.19	51.61	49.06	11.48	12.74
Ca-BDC-N=Chs	37.43	37.00	51.14	51.95	11.43	11.05
Zn-BDC-N=Chs	36.67	38.20	51.70	51.11	11.63	10.69
Ca-Zn-BDC-NH_2_	38.79	38.39	50.07	49.54	11.14	12.06
Ca-Zn-BDC-N=Chs	37.30	38.06	50.98	51.02	11.73	10.91

*Chs* Chitosan

#### Fatty acid content in seed yield

The seeds of the two quinoa genotypes, 1RE and 3YE, cultivated in Ras Sudr under saline conditions, had their oil extracted. The oil’s fatty acid content and quality were determined. Quinoa oil contains 13 fatty acids, including 5 saturated fatty acids (palmitic, stearic, arachidic, behenic, and lignoceric), and 8 unsaturated fatty acids (including 4 monounsaturated fatty acids (oleic, *cis*-11-eicosenoic, erucic, and nervonic) and 4 polyunsaturated fatty acids (linoleic, linolenic, cis-11,14-eicosadienoic and cis-13,16-docosadienoic), according to the results tabulated in Tables 9 and 10. According to the information in Tables 9 and 10, the 3YE and 1RE genotype oils had respective percentages of saturated fatty acids of 16.38% and 10.79% and unsaturated fatty acid percentages of 83.6% and 89.22%. The rate of saturated fatty acids decreased and the percentage of unsaturated fatty acids increased as a result of external spraying treatments with Ca-BDC-NH_2,_ Zn-BDC-NH_2,_Chs, Ca-BDC-N=Chs, Zn-BDC-N=Chs, Ca-Zn-BDC-NH_2,_ and Ca-Zn-BDC-N=Chs. With a 19.04% decrease in saturated fatty acid level, the Zn-BDC-NH_2_ spray had the least effect on the 3YE genotype. On the other hand, the Ca-Zn-BDC-N=Chs spray, reduced saturated fatty acids by 36.2%, which was the largest reduction. In the same 3YE genotype, the Ca-Zn-BDC-N=Chs spray compared to salt-stressed plants, improved polyunsaturated fatty acid content by 7.13%. When compared to plants under salt stress, the Zn-BDC-NH_2_ spray had the least effect on the amount of polyunsaturated fatty acids, increasing it by only 3.78%. According to the findings in the table, the Ca-Zn-BDC-N=Chs spray and Ca-BDC-N=Chs were the most effective spray treatments for raising the proportion of polyunsaturated fatty acids to saturated fatty acids in the same 3YE genotype by 8.57% and 8.52%, respectively. External spray treatments resulted in a modest decrease in saturated fatty acids and an increase in unsaturated fatty acids in the genotype 1RE, according to the findings reported in Table 10. Ca-Zn-BDC-N=Chs and Ca-BDC-N=Chs were the spray treatments that most effectively decreased saturated fatty acids and raised unsaturated fatty acids. Ca-BDC-NH_2_ spray was the spray treatment that had the least effect on saturated and unsaturated fatty acids in the 1RE genotype.

**Table 9:** Effect of external spray on fatty acids content in the crushed seed of the 3YE quinoa genotype grown under salt stress

External applications	Fatty acids composition (%)															
	Saturated					TS	Unsaturated								TUS	TUS: TS
	Palmitic	Stearic	Arachidic	Behenic	Lignoceric		Oleic	Linoleic	Linolenic	cis-11-Eicosenoic	cis-11,14-Eicosadienoic	Erucic	cis-13,16-Docosadienoic	Nervonic		
	C_16:0_	**C**_**18:0**_	**C**_**20:0**_	**C**_**22:0**_	**C**_**24:0**_		**C**_**18:1**_	**C**_**18:2**_	**C**_**18:3**_	**C**_**20:1**_	**C**_**20:2**_	**C**_**22:1**_	**C**_**22:2**_	**C**_**24:1**_		
Control	14.29	0.3	0.25	1.36	0.18	**16.38**	17.44	55.47	7.8	1.22	0.11	1.27	0.13	0.16	**83.6**	**5.1:1**
Ca-BDC-NH_2_	8.8	1.37	0.32	0.57	0.61	**11.67**	19.07	58.16	7.78	1.42	0.12	1.54	0.13	0.11	**88.33**	**7.57:1**
Zn-BDC-NH_2_	11.87	0.41	0.27	0.53	0.18	**13.26**	19.67	56.29	7.57	1.35	0.07	1.48	0.15	0.18	**86.76**	**6.54:1**
Chs	9.09	0.47	0.31	0.56	0.22	**10.65**	20.01	57.86	7.71	1.58	0.11	1.56	0.37	0.17	**89.26**	**8.38:1**
Ca-BDC-N=Chs	8.99	0.42	0.32	0.57	0.2	**10.5**	20.45	58.13	7.67	1.4	0.08	1.5	0.11	0.17	**89.51**	**8.52:1**
Zn-BDC-N = Chs	9.07	0.45	0.27	0.59	0.21	**10.59**	20.78	57.62	7.53	1.42	0.17	1.57	0.15	0.17	**89.41**	**8.44:1**
Ca-Zn-BDC-NH_2_	10.96	0.40	0.31	0.39	0.19	**12.25**	19.21	58.53	7.17	1.11	0.12	1.31	0.18	0.12	**87.75**	**7.16:1**
Ca-Zn-BDC-N=Chs	8.98	0.42	0.29	0.55	0.21	**10.45**	20.55	58.03	7.62	1.41	0.12	1.51	0.14	0.18	**89.56**	**8.57:1**

Fatty acids in the table were arranged (ascending) according to the retention time of fatty acids, which were separated from a column of the GC apparatus

*TS* Total saturated, *TUS* Total unsaturated, *Chs* Chitosan

**Table 10:** Effect of external spray on fatty acids content in the crushed seed of the 1RE quinoa genotype grown under salt stress

External applications	Fatty acids composition (%)															
	Saturated					TS	Unsaturated								TUS	TUS: TS
	Palmitic	Stearic	Arachidic	Behenic	Lignoceric		Oleic	Linoleic	Linolenic	cis-11-Eicosenoic	cis-11,14-Eicosadienoic	Erucic	cis-13,16-Docosadienoic	Nervonic		
	C_16:0_	**C**_**18:0**_	**C**_**20:0**_	**C**_**22:0**_	**C**_**24:0**_		**C**_**18:1**_	**C**_**18:2**_	**C**_**18:3**_	**C**_**20:1**_	**C**_**20:2**_	**C**_**22:1**_	**C**_**22:2**_	**C**_**24:1**_		
Control	9.3	0.41	0.3	0.56	0.22	**10.79**	19.56	58.44	7.79	1.4	0.19	1.5	0.15	0.19	**89.22**	**8.27:1**
Ca-BDC-NH_2_	9.06	0.42	0.35	0.66	0.21	**10.7**	19.18	58.76	8.09	1.36	0.1	1.48	0.15	0.18	**89.3**	**8.35:1**
Zn-BDC-NH_2_	9.22	0.43	0.29	0.56	0.15	**10.65**	18.32	59.62	8.06	1.38	0.16	1.52	0.16	0.11	**89.33**	**8.39:1**
Chs	9.09	0.42	0.23	0.55	0.21	**10.5**	19.37	58.71	8.11	1.32	0.11	1.51	0.16	0.19	**89.48**	**8.52:1**
Ca-BDC-N=Chs	8.97	0.4	0.29	0.53	0.21	**10.4**	19.83	58.73	7.7	1.39	0.12	1.49	0.15	0.19	**89.6**	**8.61:1**
Zn-BDC-N=Chs	9.0	0.44	0.32	0.51	0.22	**10.49**	20.25	58.24	7.58	1.41	0.19	1.55	0.12	0.18	**89.52**	**8.53:1**
Ca-Zn-BDC-NH_2_	9.18	0.44	0.31	0.57	0.12	**10.62**	19.06	58.91	8.11	1.40	0.12	1.50	0.14	0.14	**89.38**	**8.42:1**
Ca-Zn-BDC-N=Chs	8.96	0.31	0.26	0.56	0.21	**10.3**	20.09	58.41	7.86	1.4	0.12	1.52	0.11	0.19	**89.7**	**8.71:1**

Fatty acids in the table were arranged (ascending) according to the retention time of fatty acids, which were separated from a column of the GC apparatus

*TS* Total saturated, *TUS* Total unsaturated, *Chs* Chitosan

#### Unsaponifiable compounds in seed yield

Gas chromatography and mass spectrometry were used to study the unsaponifiables in the seed oil of two types of quinoa (1RE and 3YE) grown in saline conditions and under the influence of spray treatments of Ca-BDC-NH_2,_ Zn-BDC-NH_2,_Chs, Ca-BDC-N=Chs, Zn-BDC-N=Chs, Ca-Zn-BDC-NH_2,_ and Ca-Zn-BDC-N=Chs. Data displayed in Table 11 were found to contain a wide range of compounds, particularly long-chain fatty alcohols like 1-tetracosanol, 1-hexacosanol, and 1-octacosanol, as well as hydrocarbons, including saturated and unsaturated hydrocarbons like nonacosane and squalene, cholesterol analogs like epicholesterol, and a sizable group of phytosterols like campesterol, stigmasterol, ergostadienol acetate-7,22, beta.-sitosterol, stigmast-7-en-3-ol, (3beta,5alpha), cycloartenol and citrostadienol. Except for the Ca-Zn-BDC-N=Chs treatment, all spray treatments caused 1-tetracosanol to vanish from the 3YE genotype as compared to the control, according to the data in Table 11. In contrast, every spray treatment caused 1-hexacosanol to develop in the 3YE genotype, while every spray treatment aside from the Ca-Zn-BDC-N=Chs treatment caused the percentage of 1-hexacosanol in the 1RE genotype to drop in comparison to the control. Except for the Zn-BDC-N=Chs treatment, all spray treatments increased the percentage of the fatty alcohol1-octacosanol in the 3YE genotype relative to the control. On the other hand, the percentage of fatty alcohol 1-octacosanol decreased with all spray treatments, except for the Ca-Zn-BDC-NH_2_ treatment in the 1RE genotype compared with the control. When compared to the control, the unsaturated hydrocarbon (squalene) content of the two quinoa genotypes under study decreased after each spray treatment. In comparison to the control, spray treatments with Zn-BDC-N=Chs, Ca-Zn-BDC-NH_2,_ and Ca-Zn-BDC-N=Chs caused saturated hydrocarbon (nonacosane) to form in the 1RE and 3YE genotypes, while nonacosane appeared in the 3YE genotype following Ca-BDC-N=Chs spraying and in the 1RE genotype following Zn-BDC-NH_2_ spraying. In the same situation, the 3YE genotype showed signs of epicholesterol in comparison to the control after receiving spray treatments with Chs, Ca-BDC-N=Chs, and Ca-Zn-BDC-N=Chs. Except for Zn-BDC-NH_2_ spraying, all spray treatments resulted in campesterol production in 1RE quinoa genotype. Similarly, the phytosterol (stigmasterol) was produced in both 3YE and 1RE quinoa varieties as a result of spraying a Ca-BDC-N=Chs, Zn-BDC-N=Chs, Ca-Zn-BDC-NH_2,_ and Ca-Zn-BDC-N=Chs, while stigmasterol was produced in the 3YE variety as a result of spraying with Ca-BDC-NH_2,_ and Zn-BDC-NH_2_. Stigmasterol was produced in the 1RE variety after spraying with Chs, compared to the untreated control. Except for the Zn-BDC-NH_2_ spray treatment with the 1RE genotype, all spray treatments raised the ergostadienol acetate-7,22 content in both quinoa genotypes as compared to the untreated reference. Except for the 1RE genotype following Ca-BDC-NH_2_ spraying, all spray treatments raised the β-sitosterol level in both 3YE and 1RE quinoa varieties when compared to the control. Except for the Ca-BDC-N=Chs spray treatment with the 3YE genotype in comparison to the control, all spray treatments increased the stigmast7-en-3-ol, (3beta, 5alpha) content of quinoa seed oil in both 3YE and 1RE quinoa varieties. Additionally, the Ca-Zn-BDC-N=Chs spray treatment formed cycloartenol in the seed oil of both quinoa genotypes. In comparison to the unsprayed control, the 1RE quinoa variety produced citrostadienol in response to all spray treatments.

**Table 11:** Effect of external spray treatments on the percentage of unsaponifiable compounds (area percent %) in the milled seeds of two quinoa varieties (3YE and 1RE) grown under salt stress conditions

Unsaponifiable compounds		External applications															
		Control		Ca-BDC-NH_2_		Zn-BDC-NH_2_		Chs		Ca-BDC-*N*=Chs		Zn-BDC-*N*=Chs		Ca-Zn-BDC-NH_2_		Ca-Zn-BDC-*N*=Chs	
		3YE	1RE	3YE	1RE	3YE	1RE	3YE	1RE	3YE	1RE	3YE	1RE	3YE	1RE	3YE	1RE
Fatty Alcohol	**1-Tetracosanol [C24H50O]**	0.11	nd	nd	nd	nd	nd	nd	nd	nd	nd	nd	nd	nd	nd	0.11	nd
	**1-Hexacosanol [C26H54O]**	nd	4.19	1.82	1.63	1.29	1.63	2.12	0.87	1.33	3.2	0.62	2.29	1.67	1.59	1.38	4.39
	**1-Octacosanol [C28H58O]**	0.99	6.95	2.36	4.09	1.62	6.27	3.38	1.27	4.98	3.73	0.84	2.96	1.98	7.62	2.83	5.18
Hydrocarbon	**Squalene [C30H50]**	76.53	63.9	56.02	63.74	61.79	53.25	68.99	55.48	56.53	50.12	63.88	57.47	60.86	59.54	59.72	46.49
	**Nonacosane [C29H60]**	nd	nd	nd	nd	nd	0.11	nd	nd	1.0	nd	0.12	0.12	0.11	0.10	0.18	0.13
stereoisomer	**Epicholesterol [C27H46O]**	nd	nd	nd	nd	nd	nd	0.11	nd	0.17	nd	nd	nd	nd	nd	0.16	nd
phytosteroids	**Campesterol [C28H48O]**	nd	nd	nd	0.1	nd	nd	nd	0.11	nd	0.11	nd	0.11	nd	0.15	nd	0.14
	**Stigmasterol [C29H48O]**	nd	nd	0.11	nd	0.1	nd	nd	0.11	0.11	0.11	0.12	0.1	0.12	0.11	0.12	0.11
	**Ergostadienol acetate-7**,**22 [C28H48O]**	0.18	0.17	0.22	0.21	0.22	0.12	0.19	0.21	0.28	0.23	0.22	0.24	0.24	0.23	0.21	0.35
	**beta.-Sitosterol [C29H50O]**	3.13	6.34	10.09	5.89	8.01	9.36	5.49	8.95	9.49	11.74	7.6	10.58	9.52	8.41	6.9	13.95
	**Stigmast-7-en-3-ol**,** (3beta**,**5alpha)** **[C29H50O]**	19.07	18.45	29.38	24.25	26.98	23.41	19.73	32.82	17.95	30.59	26.6	26.03	25.51	22.13	27.22	28.84
	**Cycloartenol [C30H50O]**	nd	nd	nd	nd	nd	5.74	nd	nd	8.16	nd	nd	nd	nd	nd	1.01	0.16
	**Citrostadienol [C30H50O]**	nd	nd	nd	0.1	nd	0.11	nd	0.17	nd	0.16	nd	0.12	nd	0.12	0.17	0.26

### Phenologicalstages of quinoa genotypes (3YE and 1RE)

**Fig. 14:**
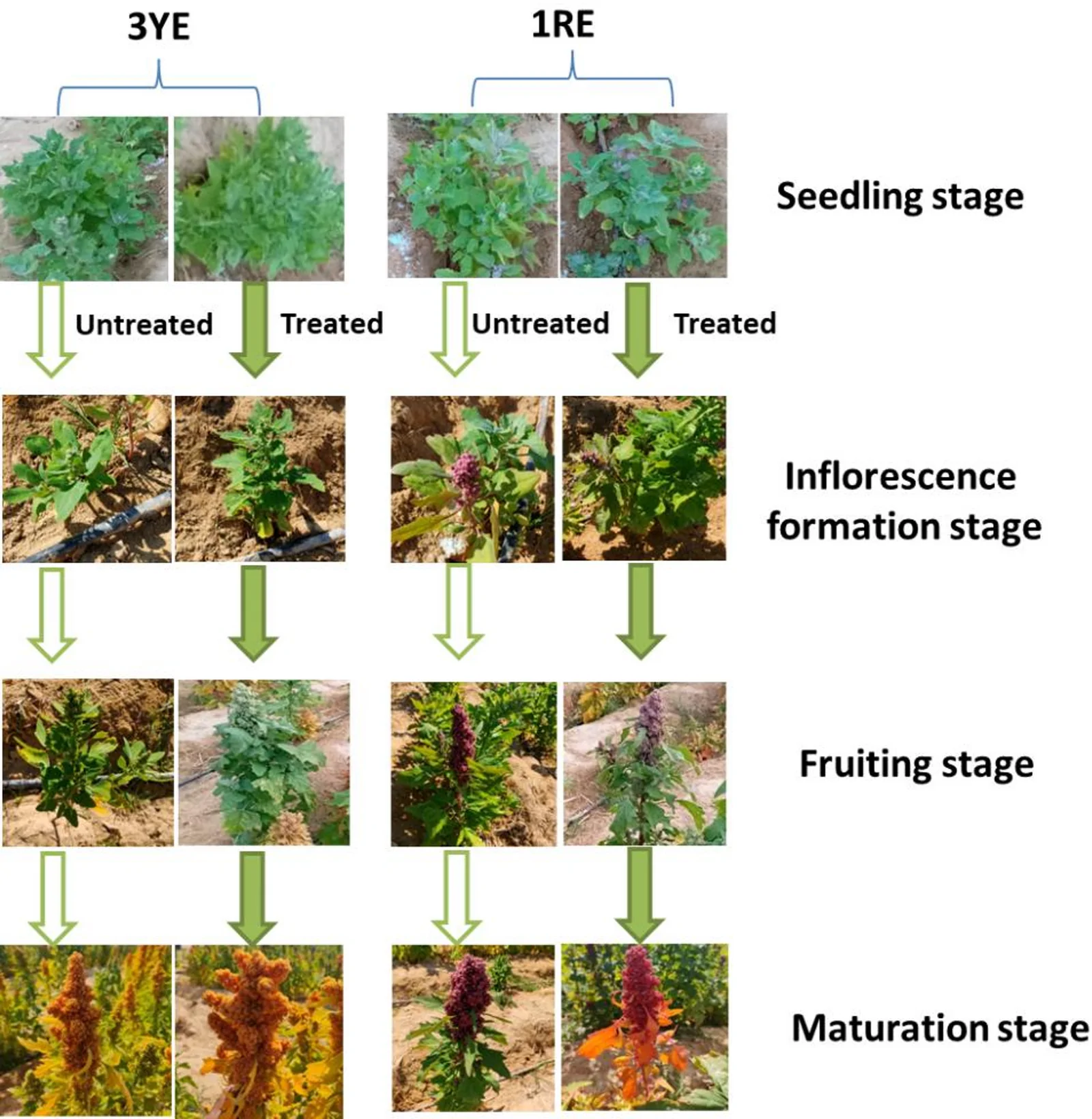
Chronological growth stages of quinoa (*Chenopodium quinoa* Willd.) genotypes 3YE and 1RE under high salinity conditions. The sequence illustrates the life cycle from seedling establishment, through inflorescence formation, flowering, fruiting, and physiological maturity (showing distinct yellow and crimson-purple phenotypes), to senescence and harvest readiness

The developmental progression of the two quinoa genotypes under saline field conditions is summarized in Fig. 14. The life cycle was characterized by the following six distinct stages: [a] Seedling Development and Establishment. Initial seedling formation (Fig. 14) confirmed the high viability and salt tolerance of both genotypes. The 3YE genotype exhibited a germination rate of 75–80%, slightly outperforming the 1RE genotype, which ranged from 70 to 75%. During this phase, the expansion of the primary stems and leaves was followed by the emergence of lateral branches, indicating successful establishment in the saline environment. [b] Inflorescence Formation: As illustrated in Fig. 14, the transition to the reproductive phase began with the development of pediculate inflorescences. These structures initially emerged on the main stems, shielded by surrounding leaves, before subsequently developing on the subordinate lateral stems. [c] Flowering Phase: The flowering stage involved the anthesis of primary inflorescence flowers for both 3YE and 1RE genotypes (Fig. 14). This stage concluded once the development of the floral organs across the panicle was complete. [d] Fruiting Stage: The fruiting phase (Fig. 14) was marked by the visible development of grains along the main stem. This period represents the critical transition from flowering to grain filling. [e] Maturation Stage: Physiological maturity was observed approximately 90 days post-planting. Initially, the grains reached a “milk stage,” characterized by a green pericarp (fruiting coat) and a sticky white internal substance that was easily crushed. By the end of this stage, the grains hardened, achieved their characteristic size, and became resistant to mechanical pressure. Distinct phenotypic color changes were noted at this stage. At the 3YE genotype, the entire plant—including leaves, stems, and fruits-transitioned to a yellow hue. At the 1RE genotype, the plant exhibited a striking transition to a crimson-purple color. [f] Senescence and Harvest: the senescence phase began with the chlorosis and subsequent necrosis of the lower leaves, followed by the browning of the stem. The total duration from planting to harvest maturity varied by genotype, 3YE was completed its cycle in 120 days. However, 1RE required a longer duration of 135 days.

### Scanning Electron Microscopy (SEM) observations

**Fig. 15:**
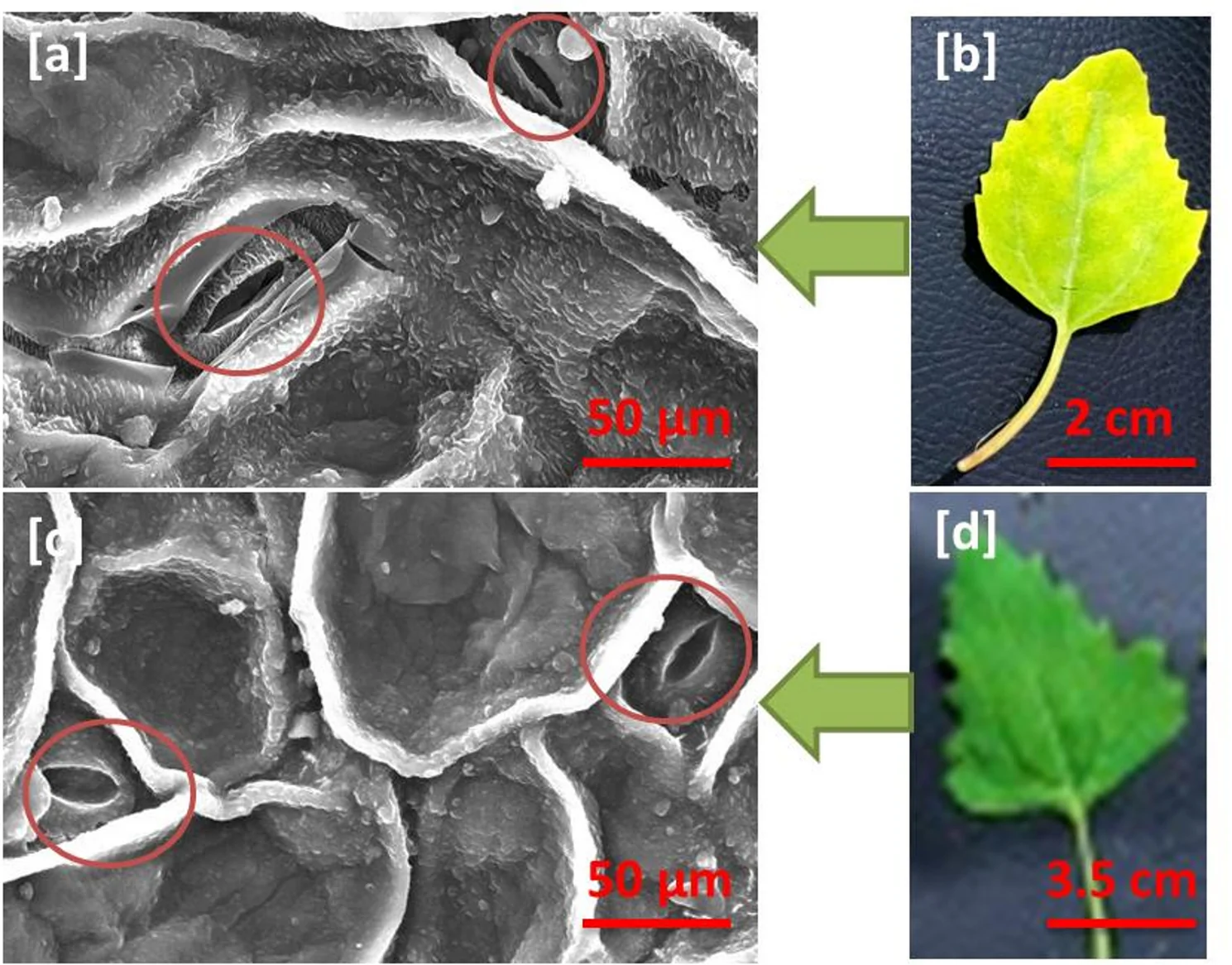
SEM micrographs showing the comparative anatomy of quinoa under salt stress. (**a**-**d**) Leaf surface demonstrating stomatal closure in control vs. stomatal opening in Chs-MOF- treated plants

**Fig. 16:**
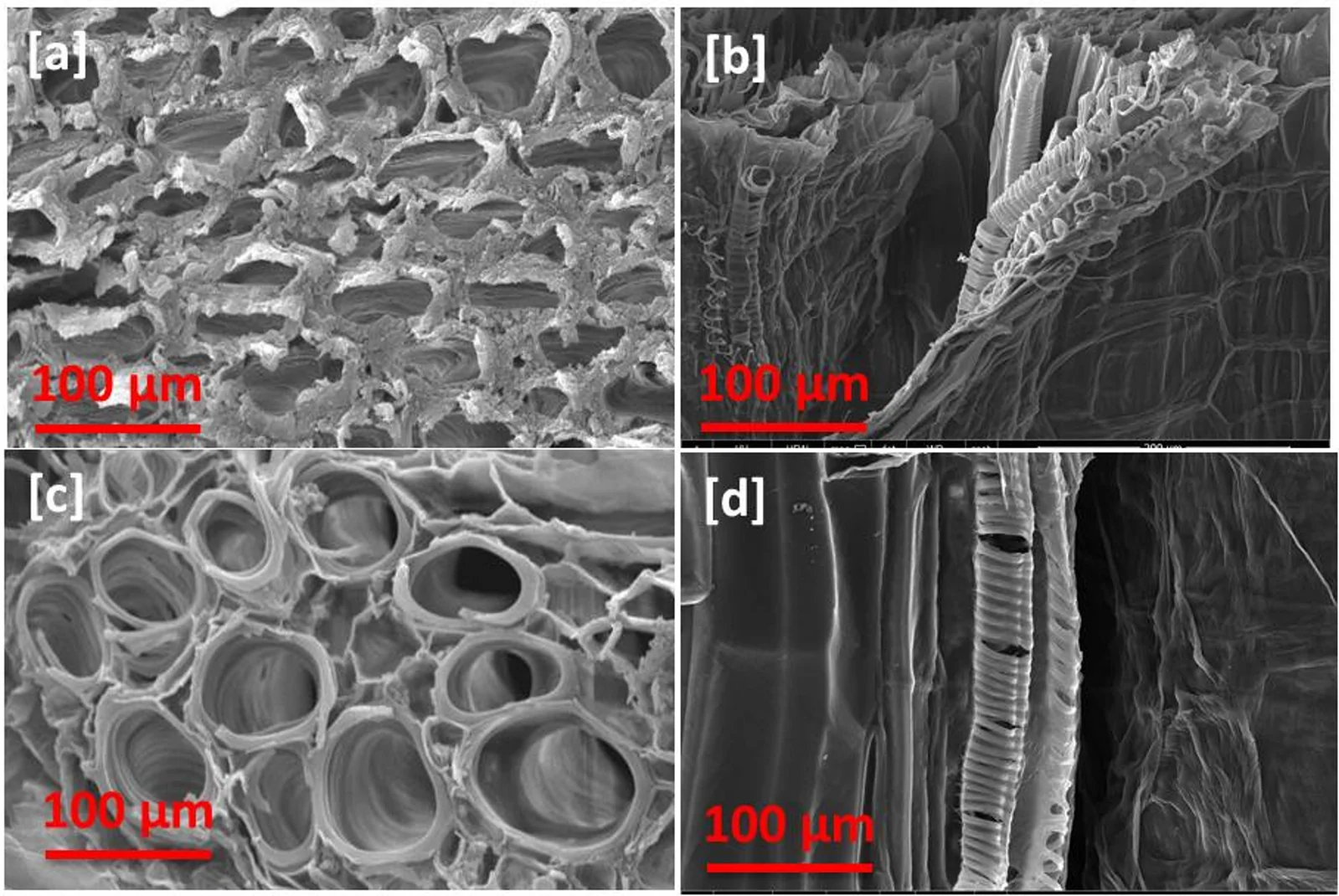
Scanning Electron Microscopy (SEM) analysis of quinoa stem architecture. Panels (**a**–**b**) highlight the adverse effects of salinity, characterized by narrowed vascular tissues and cellular atrophy. Panels (**c**–**d**) demonstrate the biostimulatory effect of the chitosan-modified framework, resulting in significantly larger, more organized xylem and phloem clusters and a robust cambium layer

Scanning Electron Microscopy (SEM) was employed to qualitatively evaluate the impact of salinity and the chemical formula of the chitosan-encapsulated Zn/Ca-MOF treatments on the micro-morphology of quinoa leaves and stems (Figs. 15 and 16). Under saline conditions, SEM analysis of the leaf epidermis revealed stomatal closure. Conversely, SEM micrographs of plants treated with the foliar-applied chitosan/MOF biostimulant revealed stomata with visibly unsegmented or open pore apertures. This visual observation indicates that the treatment helped maintain a favorable stomatal conformation under stress. Furthermore, stem cross-sections revealed that foliar biostimulant application preserved the structural integrity of the vascular bundles. In contrast to the distorted or collapsed vascular tissues observed in the salt-stressed control, biostimulant-treated plants exhibited visibly intact cell walls and well-defined vascular organization.

- A. Leaf micromorphology and stomatal features.

Under saline conditions, SEM analysis of the leaf epidermis revealed stomatal closure (Fig. 15). This response is typically associated with a defense mechanism to prevent water loss, though it visually alters the surface characteristics of the epidermis. Conversely, leaf surfaces treated with the foliar chitosan/MOF biostimulant showed stomatal pores with a predominantly open morphology under SEM. This visual observation aligns with the higher chlorophyll accumulation and improved phenotypic appearance of the foliage.

- B. Stem anatomical structure (cross and longitudinal sections).

Under salt stress, cross-sections of quinoa stems revealed pronounced tissue disruption, characterized by collapsed parenchymal cells and distorted vascular bundles (Fig. 16). Longitudinal sections revealed a visibly narrowed and less distinct cambium layer compared to the normal tissue organization. Following the application of the Chs-MOF biostimulant, a distinct visual mitigation of tissue damage was evident within the vascular system. Longitudinal sections revealed broad, well-defined, and continuous xylem and phloem vessels, demonstrating intact cell wall morphology and uncollapsed lumen dimensions under saline field conditions.

- C. Potential mechanism of biostimulation.

Chitosan is widely documented to stimulate the endogenous synthesis of key growth regulators, particularly auxins and gibberellins, which drive cell elongation and tissue expansion. The presence of Zn^2+^ and Ca^2+^ on the framework matrix promotes efficient nutrient translocation and safeguards cellular integrity. Concurrently, these coordinated biochemical responses enhance the morphological adaptability of plants exposed to hypersaline conditions.

## Discussion

Foliar application of the Chs-MOF composite significantly improved vegetative growth and biomass production under saline conditions. This protective efficacy stems from the active functional moieties of the chitosan support combined with zinc and calcium on the core framework. These biostimulants and nutrients helped maintain photosynthetic capacity and tissue growth under saline field conditions [69]. Calcium eventually contributes to improving growth and yield in saline circumstances by regulating ion uptake, maintaining cell turgor, and guarding against the detrimental effects of salt stress [70, 71]. Zinc helps quinoa plants combat the detrimental effects of salinity. In addition to being a micronutrient, zinc is essential for enhancing the absorption of vital nutrients, such as copper, manganese, and iron [72]. Additionally, zinc helps to lessen the absorption of heavy metals like lead and cadmium, which can be problematic in saline soils. Zinc lowers oxidative damage and increases the activity of antioxidant enzymes and the carbonic enzyme necessary for the production of chlorophyll [73, 74]. Additionally, zinc increases water content, stomatal conductivity, cell division, and cell membrane stability, all of which enhance plant performance in saline conditions [75]. In quinoa roots, zinc has been reported to influence the expression of transcription factors and metal transport genes, which could play a role in modulating the plant’s capacity to withstand salinity [76]. Maintaining adequate Zinc levels via foliar application likely contributed to improved stress tolerance under saline conditions. Because Zinc plays an established role as a cofactor in phytohormone regulation and protein synthesis [76], supplying bioavailable Zn via the hybrid formulation supported metabolic function, consistent with the observed maintenance of growth parameters under saline field conditions, this may explain the enhanced growth characteristics observed in our study, though the underlying molecular pathways remain to be directly evaluated. Quinoa’s biomass can be increased and its tolerance to salt stress improved with the help of chitosan. As a biostimulant, it enhances physiological responses and encourages plant growth in salinity-prone environments. In particular, chitosan can boost antioxidant defense systems [23, 77], promote root and shoot growth, and help plants better control ions and produce osmoprotectants [78]. Because soluble sugars, proteins, and proline are compatible solutes, chitosan can raise their levels, assisting plants in maintaining osmotic balance when exposed to salt stress [79].

In the Ras Sudr region of South Sinai, Egypt, elevated soil salinity exacerbated oxidative stress in plants. This triggered an overproduction of reactive oxygen species (ROS) under these hostile growing conditions, ultimately leading to a decline in both quinoa grain and straw yields [31]. Quinoa yields have increased as a result of the use of new technologies, such as spraying with Ca-BDC-NH_2_, Zn-BDC-NH_2_, Chs, and Ca-Zn-BDC-N = Chs. This is in line with earlier research [80, 81], that found in salty soils, foliar treatment of Ca, Zn, and Chs can enhance quinoa seed and straw yield. Zinc is also necessary for the correct operation of numerous enzymes involved in plant metabolism, such as those that synthesize proteins, carbohydrates, nucleic acids, and chlorophyll, all of which are important for the productivity and health of quinoa plants [82].

Elevated salt concentrations can severely impair photosynthetic pigments. This typically manifests as a reduction in chlorophyll content, disruption of photosystems, and a subsequent decline in overall photosynthetic efficiency [83]. According to the study’s results, quinoa leaves grown in saline media had better photosynthetic pigments and more chlorophyll when zinc and calcium were loaded onto metal-organic frameworks, as well as chitosan and their combinations. These findings are entirely in line with earlier research [84–86]. This effect is frequently linked to calcium, which plays an important role in maintaining healthy cell structures and promoting efficient photosynthetic processes, potentially protecting against abiotic stresses [87]. Additionally, zinc serves as a cofactor for many of the enzymes that are involved in photosynthesis. Furthermore, zinc is necessary for the proper operation of photosynthetic enzymes, such as those that fix carbon dioxide [88]. Likewise, chitosan increases photosynthesis’ efficiency by increasing the amount of light energy needed to complete the process [23].

The growth of quinoa was adversely affected by salinity, which increased oxidative stress on quinoa plants [17], which also caused the loss of selective permeability of the plasma membranes and ionic toxicity from the buildup of salts and free radicals in the tissues. This toxicity speeds up leaf senescence and hinders the absorption of nutrients. These findings are entirely in line with earlier studies on the quinoa plant [18, 19]. On the other hand, free radical accumulation was decreased, and H_2_O_2_ and MDA levels were decreased when chitosan, zinc, and calcium were sprayed on quinoa seedlings. This effect suggests that chitosan enhances the plants’ antioxidant defense system, reducing oxidative damage and improving tolerance to saline stress by increasing antioxidant enzyme activity such as POD and SOD, which help neutralize harmful free radicals and reactive oxygen species [89]. Chitosan also increases the production of beneficial metabolites, such as glutathione and α-tocopherol, which further contribute to the plant’s defense against oxidative stress [79]. Furthermore, chitosan aids in preserving the cellular redox equilibrium of the plant by regulating the amounts of ROS and antioxidants [90]. Likewise, zinc reduces indicators of oxidative damage like H_2_O_2_, a reactive oxygen species, and MDA, an indicator of lipid peroxidation, and enhances quinoa physiological activity to better withstand stressful conditions [46]. Additionally, foliar spray treatment of calcium on quinoa leaves, which can enhance membrane stability, boost the synthesis of protective substances such as proline and sugars that aid in the plant’s adaptation to environmental stressors, reduce oxidative damage, and significantly decrease free radical formation, all of which safeguard the plant’s overall health and development [91]. The poor activity of antioxidant enzymes (SOD and POD) in quinoa plants grown under severe saline environments is confirmed by findings obtained earlier. The outcomes are consistent with previous research [92]. It was found that because of the buildup of free radicals and an imbalance in the defensive systems, salinity might hinder the plant’s defenses. Antioxidant enzyme activity is reduced as a result. It creates a great deal of oxidative stress, which seriously damages plant tissues. Quinoa seedlings treated with chitosan, zinc, and calcium had an overall increase in antioxidant capacity by boosting endogenous antioxidant systems and raising the activity of antioxidant enzymes like POD and SOD [93]. A well-known natural and eco-friendly substance, chitosan helps plants defend themselves against stress and keeps their defense system in balance. By removing free radicals and chelating metal ions, chitosan also has antioxidant action. Chitosan contributes to the preservation of cell membrane integrity and functions by enhancing the equilibrium between the generation and elimination of reactive oxygen species [94]. Additionally, quinoa plants’ antioxidant enzyme activity is enhanced by zinc spraying. Zinc serves as an indispensable cofactor for the conformational stability and catalytic activity of key antioxidant enzymes, including superoxide dismutase (SOD), catalase (CAT), and ascorbate peroxidase (APX), thereby facilitating the scavenging of reactive oxygen species (ROS) [95]. These enzymes directly eliminate reactive oxygen species (ROS) that cause oxidative damage to cells [96]. In the same direction, Calcium application on quinoa plants enhanced the activities of SOD, catalase, and ascorbic acid peroxidase. While keeping hydrogen peroxide levels low [97].

While the total amino acid yield increased under treatments like Ca-Zn-BDC-N=Chs, the relative shifts in individual amino acid percentages were modest. Interestingly, the percentage of essential amino acids slightly decreased, while non-essential amino acids increased.

Modification of chitosan with metal–organic frameworks has been shown to enhance the accumulation of total amino acids—most notably lysine—in quinoa seeds cultivated under saline conditions. This enrichment aligns with earlier reports highlighting the role of amino acid biosynthesis in osmoregulation and storage protein synthesis under salinity [98, 99]. The spray chemicals Ca-BDC-NH_2_, Zn-BDC-NH_2_, Chs, and Ca-Zn-BDC-N=Chs may be responsible for the beneficial effect on raising the amino acid content in quinoa seeds cultivated in saline circumstances. Calcium, which aids in the improved absorption of other minerals by plants, particularly those involved in the synthesis of amino acids, is necessary for these benefits [100]. The metabolic processes involved in the production of amino acids can be affected by calcium [101]. According to studies, quinoa seeds’ amounts of particular amino acids may alter as a result of salt. Proline, for instance, may rise in saline media to facilitate osmotic adjustment, while other amino acids might experience it. In order to improve the balance of amino acid levels, calcium can help attenuate these fluctuations [101]. Furthermore, quinoa seeds’ amino acid composition can be preserved or even enhanced with zinc. The reason for this is that a plant that is healthier and less stressed by salinity will be able to produce and accumulate a greater variety of amino acids [102]. In a similar vein, chitosan spraying can enhance the amino acid content of quinoa plants, particularly those cultivated in salty soils. It has been demonstrated that chitosan enhances plant growth, nutrient absorption, stress tolerance, and protein synthesis-all of which may have an indirect effect on the synthesis of amino acids [78]. Chitosan may be able to enhance the amino acid content of quinoa seeds by mitigating the detrimental effects of salt stress in saline environments. Increased synthesis and storage of amino acids, especially in the roots and seeds, can result from chitosan’s effects on nitrogen metabolism and transport throughout the plant [103].

The exogenous application of metal-organic frameworks (Ca-, Zn-, and Ca-Zn-BDC-NH_2_) and their chitosan composites (Chs and Schiff bases) effectively modulated the fatty acid composition of both quinoa genotypes under saline field conditions. Notably, the Ca-Zn-BDC-N=Chs spray exhibited the highest efficacy, reducing saturated fatty acids (SFAs) by up to 36.2% and improving polyunsaturated fatty acids (PUFAs) by 7.13% in the 3YE genotype. However, when evaluating these findings, it is essential to distinguish between statistical significance and practical nutritional relevance. Quinoa oil inherently possesses a highly favorable nutritional profile, characterized by high unsaturated fatty acid fractions (83.6% in 3YE and 89.22% in 1RE). While the treatments successfully minimized the adverse effects of salt stress by shifting the fatty acid balance toward higher unsaturation, the absolute baseline of these healthy fats remained robustly high across all groups. From a dietary perspective, the modest percentage increases in PUFAs (ranging from 3.78% to 7.13%) and the slight shifts in the PUFA/SFA ratios do not fundamentally alter the macronutrient value or the dietary category of the oil. Instead, these changes signify a modest metabolic stabilization induced by the treatments, protecting the seed’s lipid quality against oxidative salinity stress without drastically shifting its core functional or health-promoting properties. Therefore, the basic nutritional profile of both the 1RE and 3YE genotypes remains stable and consistently high in quality, irrespective of the minor fluctuations driven by the treatments. Elevated soil salinity can change the content of seeds, notably the amount of fatty acids, and have a detrimental effect on plant growth. Additionally, salinity can lower the amounts of particular unsaturated fatty acids, which are critical for the nutritional value and overall health of plants. This investigation showed that various spray treatments affected the fatty acid content of quinoa seeds under saline conditions, increasing the amount of monounsaturated fatty acids like oleic and polyunsaturated fatty acids like linoleic and linolenic. These outcomes closely align with previous research [104], which demonstrated that foliar zinc applications increase linoleic acid content in quinoa seeds. Furthermore, a prior study [101] indicates that zinc spraying can help restore fatty acid equilibrium, mitigating the adverse effects of salinity. Additionally, *Brassica napus’s* fatty acid profile was enhanced by zinc oxide nanoparticles [105]. Chitosan spraying has a beneficial effect on the fatty acid content of quinoa seed. Chitosan stimulates effect on the absorption of essential minerals such as manganese, zinc, and iron, which are involved in several metabolic processes, including the creation of fatty acids [106]. Similarly, chitosan increases the activity of antioxidant enzymes like proline and catalase, which shield plants from oxidative damage brought on by salt stress [78]. Fatty acid production is indirectly aided by this protection. Furthermore, chitosan helps preserve or even raise the amounts of certain healthy fatty acids in quinoa seeds by reducing the adverse effects of salinity [103]. For instance, research has indicated that chitosan helps support the maintenance of oleic and linoleic acid levels, two fatty acids that are critical to human health [101]. In this regard, zinc is also necessary for the activity of fatty acid synthase and acetyl-CoA carboxylase, two enzymes involved in fatty acid synthesis [104]. Additionally, by reducing the detrimental effects of salinity on fatty acid synthesis, foliar zinc spraying may raise levels of healthy fatty acids like linoleic acid, an omega-6 fatty acid [105].

While foliar application of the biostimulant statistically altered the lipid and amino acid profiles of both quinoa genotypes under saline field conditions, the modest magnitude of these shifts warrants cautious interpretation. The minor increases in specific phytosterols (β-sitosterol and campesterol) and long-chain fatty alcohols indicate a preserved lipid balance and enhanced stress responsiveness, which may support membrane stabilization against salt-induced oxidative damage. Crucially, these minor fluctuations did not alter the overall nutritional architecture of the seed, allowing quinoa to maintain its baseline nutritional value despite severe salinity. Previous studies confirm that quinoa unsaponifiable lipids are rich in phytosterols (predominantly stigmasterol, β-sitosterol, and campesterol), tocopherols, and long-chain alkanes [107–109], which contribute to both structural stress tolerance in plants [110] and functional nutritional benefits [111, 112]. Furthermore, genotype-dependent variations in stress response align with their baseline physiological characteristics; the 3YE genotype exhibits a higher germination rate (75–80%) and earlier maturation (120 days post-planting) compared to the late-maturing 1RE genotype (70–75% germination, 135-day harvest).

The observed improvement in plant performance under saline field conditions is consistent with the cooperative actions of the bi-elemental Zn/Ca framework and its chitosan coating. While Zinc serves as an essential cofactor for superoxide dismutase (SOD) and chlorophyll stabilization, calcium is recognized in the literature as a vital secondary messenger involved in osmotic adjustment and ionic regulation. Although specific pathways like the SOS signaling cascade and vacuolar ion partitioning remain to be verified through direct molecular and ion-profiling assays, the foliar application of Chs@Zn/Ca-MOFs effectively supported antioxidant defenses and chloroplastic stability in treated quinoa plants [113].

The incorporation of chitosan is intended to act as a protective coating, a strategy recognized in biopolymer literature for modulating metal ion delivery and minimizing nanoparticle-induced phytotoxicity [114, 115]. While detailed dissolution kinetics and gene expression pathways (*P5CS* modulation) remain to be experimentally verified for this formulation, biopolymer-MOF composites are hypothesized to support cellular osmotic balance under saline field conditions [116]. These findings highlight the potential of multi-elemental nanocomposites as promising tools for sustainable crop management in salinized soils [116, 117].

## Conclusion

The findings of this study underscore the transformative potential of chitosan-modified di-metallic frameworks (Chs@Zn/Ca-MOFs) as a multifunctional solution for enhancing crop growth under saline field conditions. Successful synthesis of these hybrids was validated by precise analytical techniques, while biological evaluation on 3YE and 1RE quinoa genotypes demonstrated that the frameworks function as both high-efficiency fertilizers and potent biostimulants. Specifically, the di-metallic framework (Zn/Ca) demonstrated physiological and yield superiority over the mono-metallic (Zn) version. This treatment resulted in significant increases in plant length, seed yield, and photosynthetic pigment concentrations. Furthermore, the hybrids fortified the plants’ internal defense systems, as evidenced by a dramatic reduction in malondialdehyde (MDA) and hydrogen peroxide levels, alongside the simultaneous up-regulation of superoxide dismutase (SOD) and peroxidase (POD) enzymatic activities. Qualitative SEM examination revealed that the formulation helped preserve visible surface morphology and stomatal appearance under saline conditions. Overall, integrating metal-organic frameworks with chitosan provides a promising foliar biostimulant strategy to enhance quinoa performance and stress tolerance in salinized soils.

## Supplementary Information

Supplementary Material 1.

## Data Availability

The datasets used and/or analysed during the current study are available from the corresponding author on reasonable request.
