# Supplementary material for: Synthesis of chitosan-encapsulated mono- and di-metallic (Zn/Ca) MOFs to modulate seed quality and antioxidant defense in Chenopodium quinoa under salinity stress
Source: BMC Plant Biol. 2026 Aug 21;26:1444. doi: 10.1186/s12870-026-09734-w (PMC13499332; doi:10.1186/s12870-026-09734-w)
Supplement: Supplementary file 1 [file 12870_2026_9734_MOESM1_ESM.docx]

**Supporting information’s**

| **Table S1.** Chemical analysis of the experimental soil and irrigated water | | | | | | | | | | | | |
| --- | --- | --- | --- | --- | --- | --- | --- | --- | --- | --- | --- | --- |
| sample | depth | pH | EC (dSm^-1^) | EC (ppm) | Cations (meq l^-1^) | | | | Anions (meq l^-1^) | | | |
|  |  |  |  |  | Ca^2+^ | Mg^2+^ | Na^+^ | K^+^ | CO_3_^2-^ | HCO_3_^-^ | Cl^-^ | SO_4_^2-^ |
| Soil | 0-30 | 7.31 | 3.5 | 2240 | 5.27 | 12.87 | 12.23 | 1.27 | 0 | 2.96 | 18.33 | 10.33 |
| water | --- | 6.9 | 15.09 | 9657.6 | 57.75 | 13.81 | 51.88 | 1.72 | 0 | 3.49 | 91.65 | 30.12 |
